# Speciation, Protein Binding, Biotransformation, and Cytotoxicity of a V^V^–Lactate Complex

**DOI:** 10.1021/acs.inorgchem.6c02377

**Published:** 2026-08-15

**Authors:** Maddalena Paolillo, Virginia Cuomo, Giarita Ferraro, Paola Imbimbo, Nadiia I. Gumerova, Federico Pisanu, Eugenio Garribba, Annette Rompel, Antonello Merlino

**Affiliations:** † Department of Chemical Sciences, 9307 University of Naples Federico II, Complesso Universitario di Monte Sant’Angelo, via Cintia, Napoli I-80126, Italy; ‡ 27258 Universität Wien, Fakultät für Chemie, Institut für Biophysikalische Chemie, Wien 1090, Austria; § Dipartimento di Medicina, Chirurgia e Farmacia, 9312 Università di Sassari, Viale San Pietro, Sassari I-07100, Italy

## Abstract

We studied the speciation, protein binding, biotransformation, and cytotoxicity of the dioxidovanadium­(V) lactate complex Cs_2_[V^V^_2_O_4_(lact)_2_]·2H_2_O and compared the results with those obtained for the analogous malate compound. ^51^V NMR and ESI-MS results show that Cs_2_[V^V^_2_O_4_(lact)_2_]·2H_2_O forms [V^V^O_2_]^+^, [H_2_V^V^O_4_]^−^, [H_2_V^V^_2_O_7_]^2–^, [V^V^_2_O_4_(lact)_2_]^2–^, [V^V^_3_O_7_(lact)_2_]^3–^, [V^V^_4_O_12_]^4–^, [V^V^_5_O_15_]^5–^, [V^V^O_2_(lact)­(H_2_O)]^−^, [V^V^O_2_(lact)­(OH)]^2–^, and [V^V^_10_O_28_]^6–^ species in aqueous solution. In the presence of lysozyme, the amounts of [V^V^_10_O_28_]^6–^ and [V^V^O_2_(lact)­(H_2_O)]^−^ significantly decrease and protein adducts with [V^V^_2_O_4_(lact)_2_]^2–^ and [V^V^O­(lact)_2_]^−^ are detected by ESI-MS. X-ray structures of the adducts show noncovalent binding of [V^IV^O]^2+^, [V^V^O_2_]^+^, [V^V^_2_O_4_(lact)_2_]^2–^, cyclic [V^V^_3_O_9_]^3–^, and [V^V^_3_O_7_(lact)_2_]^3–^ to lysozyme. Cs_2_[V^V^_2_O_4_(lact)_2_]·2H_2_O and Cs_2_[V^V^_2_O_4_(mal)_2_]·2H_2_O exhibit higher cytotoxicity than cisplatin on PC-3 cancer cells (IC_50_ values are 5.9 ± 0.3 and 5.0 ± 0.3 μM, respectively), while they are less active than cisplatin against HeLa cells and less selective against BALB/c-3T3 and HaCaT cells. In systems containing [V^V^_2_O_4_(lact)_2_]^2–^ and biological reductants, EPR studies demonstrate the formation of hydroxyl radicals, supporting a mechanism in which redox cycling between V^V^ and V^IV^ contributes to the oxidative stress that accounts for the observed biological activity.

## Graphical abstract

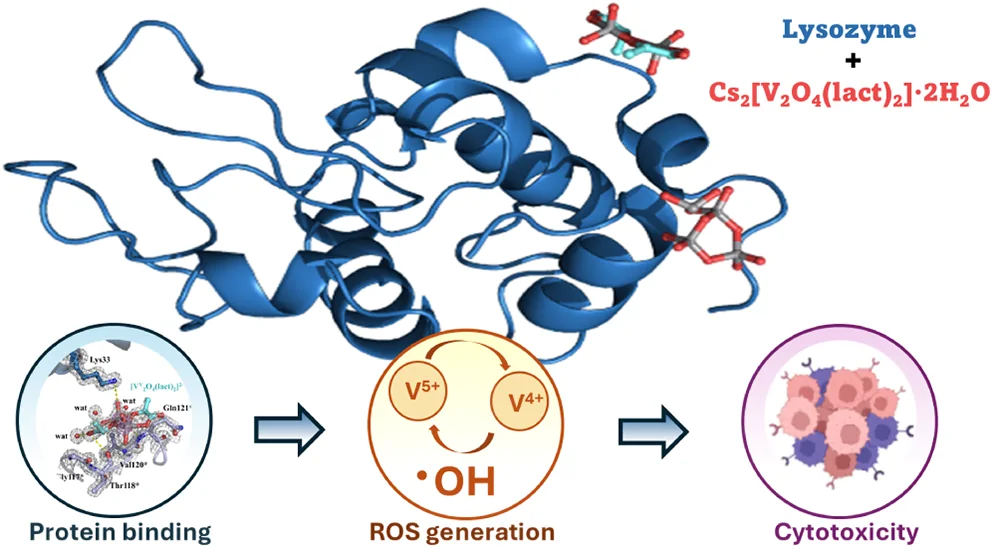

## Introduction

Vanadium is a trace element that exhibits a rich and versatile coordination chemistry in aqueous and biological environments, where it can exist in multiple oxidation states, primarily +4 and +5.^1^ This redox flexibility, combined with a strong affinity for O-donor ligands, is the basis for the wide range of biological and pharmacological activities associated with vanadium compounds (VCs). In this context, VCs have attracted considerable attention for the possible treatment of various diseases, particularly type II diabetes and some forms of cancer.^2–5^

Under physiological conditions, the speciation of vanadium depends on several variables, such as pH, concentration, redox conditions, and the presence of endogenous ligands. α-Hydroxycarboxylates such as glycolate, lactate, citrate, and malate play a particularly important role because they are abundant in biological fluids and can efficiently coordinate both V^IV^ and V^V^ centers through their carboxylate and α-hydroxy groups, forming stable five-membered chelate rings.^6–10^ As a result, these bioligands can significantly influence, *in vivo*, the transport, distribution, and reactivity of vanadium. The interaction between vanadium and α-hydroxycarboxylates is also closely related to cellular metabolism. Lactate, in particular, is present at millimolar concentrations in many tissues and plays a key role in glycolysis and cancer metabolism. Its ability to bind vanadium suggests that V-lactate species may form under physiological conditions and contribute to the intracellular speciation of vanadium. Coordination to α-hydroxycarboxylates can modulate the redox properties of VCs, affecting their biotransformation pathways and biological activity through the stabilization of V^IV^ whose complexes are more stable than the corresponding ones formed by V^V^.^11,12^ The V^V^/V^IV^ interconversion is often associated with the generation of reactive oxygen species (ROS) and with the pharmacological effects of VCs.^1–3,13^ Finally, it must be noticed that VCs of α-hydroxycarboxylates also show biological activity.^14^

A crucial aspect in the biological behavior and pharmacological action of VCs is their interaction with proteins.^15–18^ In biological fluids, vanadium species are known to bind to serum proteins such as transferrin and albumin, as well as to intracellular proteins, which can act as carriers, transporters, reservoirs, or targets. Protein binding is strongly influenced by the speciation of vanadium in solution, which—in turn—depends on the nature of the coordinating ligands. Due to their biological importance and high concentration in biological fluids, α-hydroxycarboxylate can be involved in the formation of vanadium–protein adducts, determining their structural and functional properties.

Recently, we have investigated in detail the speciation in 1.1 M sodium chloride, 0.1 M sodium acetate, pH 4.0, at room and physiological temperature, and the binding to lysozyme of a dinuclear V^V^ complex formed by malate (mal), Cs_2_[V^V^_2_O_4_(mal)_2_]·2H_2_O (Figure 1).^19^ Lysozyme has been used as a model system since it is cheap, well-folded, commercially available, stable, easy to crystallize, and can be analyzed by mass spectrometry. The results demonstrated that the compound transforms in solution forming [V^V^_2_O_4_(mal)_2_]^2–^, [V^V^_2_O_5_(mal)]^2–^, and decavanadate (V_10_). The structural data showed covalent binding of [V^IV^O]^2+^ and [V^V^_10_O_26_]^2–^ to the protein and noncovalent binding of [V^V^_2_O_5_(mal)]^2–^ and [V^V^_10_O_28_]^6–^ with surface protein residues.^19^

**Figure 1:**
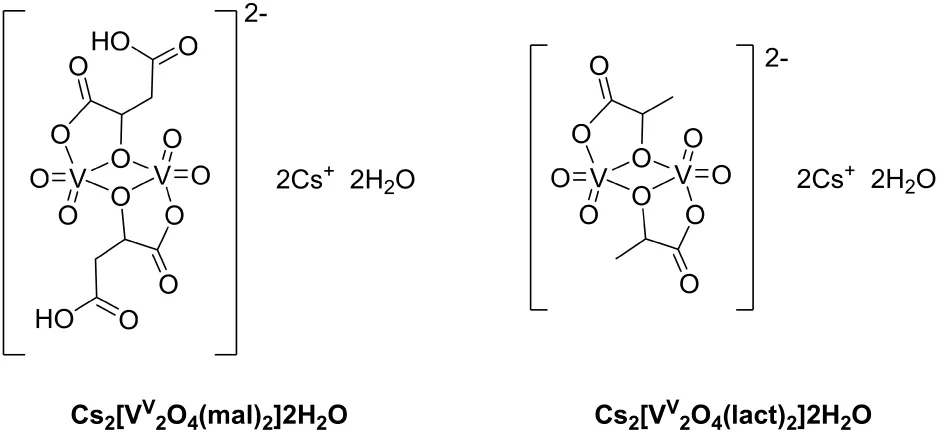
Structures of Cs_2_[V^V^_2_O_4_(mal)_2_]·2H_2_O and Cs_2_[V^V^_2_O_4_(lact)_2_]·2H_2_O.

Here we investigated the speciation, protein binding, biotransformation, and cytotoxicity of the dioxovanadium­(V) lactate complex, Cs_2_[V^V^_2_O_4_(lact)_2_]·2H_2_O (Figure 1), where lact = lactate. Its behavior was analyzed by a combined approach applying ^51^V nuclear magnetic resonance (^51^V NMR), electrospray ionization-mass spectrometry (ESI-MS), X-ray crystallography, and electron paramagnetic resonance (EPR) under different experimental conditions. The results were compared with those previously obtained for the analogous malate complex, Cs_2_[V^V^_2_O_4_(mal)_2_]·2H_2_O.^19^ This integrated approach allows us to assess how the nature of the α-hydroxycarboxylate ligand influences vanadium speciation, its interaction with proteins, and its biological activity.

## Materials and Methods

### Synthesis and Characterization of Cs_2_[V^V^_2_O_4_(lact)_2_]·2H_2_O

The synthesis of Cs_2_[V^V^_2_O_4_(lact)_2_]·2H_2_O was carried out following the procedure previously described.^20^ Briefly, V^V^_2_O_5_ (1 mmol, 0.182 g) and Cs_2_CO_3_ (1.1 mmol, 0.358 g) were mixed in water (5 mL) for 1 h at 40 °C. The solution was then cooled with ice, and lactic acid (2 mmol, 0.181 g) was added. The solid formed upon the addition of lactate was filtered, washed with a small amount of EtOH, and air-dried at RT.

Cs_2_[V^V^_2_O_4_(lact)_2_]·2H_2_O (643.84 g/mol): Anal. Calc. for C_6_H_12_Cs_2_O_12_V_2_: C, 11.19; H, 1.88; Cs_2_O + V_2_O_5_, 72.0; H_2_O, 5.6%. Found: C, 11.32; H, 1.73; Cs_2_O + V_2_O_5_, 70.0; H_2_O: 5.0%.

### ^51^V NMR Studies

^51^V NMR spectra of Cs_2_[V^V^_2_O_4_(lact)_2_]·2H_2_O (concentration = 5 mM) were collected at RT in D_2_O (as a reference), and in (i) 1.1 M sodium chloride, 0.1 M sodium acetate, pH 4.0, 10% D_2_O; (ii) 20% ethylene glycol, 0.1 M sodium acetate, pH 4.0, 0.6 M sodium nitrate, 10% D_2_O; (iii) 0.8 M succinic acid, pH 7.0, 10% D_2_O; (iv) 2.0 M sodium formate, 0.1 M HEPES, pH 7.5, 10% D_2_O. Spectra were also collected under conditions (i–iv) after 1 h and 7 days of incubation with lysozyme (concentration = 13 mg/mL, 0.9 mM). V^V^OCl_3_ was used as a reference for the chemical shifts (in parts per million (ppm) relative to δ). The ^51^V NMR spectra were obtained at 131.60 MHz with a relaxation delay of 0.01 s and an accumulation time of 0.05 s. The number of scans was 2000. To plot the ^51^V NMR spectra, MestReNova with Automatic Phase Correction and Whittaker Smoother as the baseline were used.

### Mass Spectrometry

The mass spectrometry study was performed using a timsTOF flex LC-MS System supplied by Bruker Daltonics Ltd. Spectra of Cs_2_[V^V^_2_O_4_(lact)_2_]·2H_2_O (concentration ∼ 100 μM) were acquired in H_2_O and in acetonitrile/methanol + 1% H_2_O in positive and negative ion mode within the *m*/*z* range 90–600. The spectrometer was calibrated using the standard TuneMix (Bruker Daltonics GmbH, timsTOF Pro User Manual, Bremen, Germany) to ensure an accuracy of approximately 5 ppm in the *m*/*z* region 50–1800. The deconvoluted spectra were obtained using Bruker Daltonics Data Analysis 4.0 software. Other parameters: capillary voltage: 2500 V; nebulizer: 0.3 bar; dry gas flow: 3.0 L/min (nitrogen); dry heater temperature: 200 °C; flow rate: 3 μL/min.

To collect the spectra of lysozyme, protein samples (concentration = 13 mg/mL, 0.9 mMin water) were diluted 1:100 in acetonitrile/H_2_O 9/1 + 1% formic acid (FA). The spectra were then collected in the absence and presence of Cs_2_[V^V^_2_O_4_(lact)_2_]·2H_2_O (ratio 1:1) after an incubation time of 5 d. The deconvoluted spectra were obtained using MagTran software based on the ZScore algorithm.^21^ Other settings: capillary voltage: 3500 V; nebulizer: 0.3 bar; dry gas flow: 3.0 L/min (nitrogen); dry heater temperature: 200 °C; mass range: 300–3000 *m*/*z*.

### Crystallization, Data Collection, Structure Resolution, and Refinement

The lysozyme crystals (13 mg/mL, 0.9 mM) were grown using the hanging drop vapor diffusion method under the following experimental conditions: (i) 1.1 M sodium chloride, 0.1 M sodium acetate, pH 4.0; (ii) 20% ethylene glycol, 0.1 M sodium acetate, pH 4.0, 0.6 M sodium nitrate; (iii) 0.8 M succinic acid, pH 7.0; (iv) 2.0 M sodium formate, 0.1 M HEPES, pH 7.5, as reservoirs. The crystals of the adducts with Cs_2_[V^V^_2_O_4_(lact)_2_]·2H_2_O were obtained by treating V-free lysozyme crystals with the metal compound for 7 days. The X-ray diffraction data were then collected at the ID23-2 beamline of the European Synchrotron Radiation Facility (ESRF) of Grenoble (France), except for lysozyme crystals treated with Cs_2_[V^V^_2_O_4_(lact)_2_]·2H_2_O in 1.1 M sodium chloride, 0.1 M sodium acetate, pH 4.0. These data were collected at the XRD2 beamline of the Elettra synchrotron of Trieste (Italy), at 100 K. Crystals were cryoprotected with a solution containing 75% reservoir and 25% glycerol. Data were processed with the Global Phasing autoPROC pipeline.^22^ Data collection statistics are reported in Table S1 of the Supporting Information (SI).

The phase problem was solved in all cases by molecular replacement using Phaser,^23^ with the coordinates from PDB code 193L^24^ as a starting model. The structures were isotropically refined using Refmac5.^25^ Vanadium positions were identified by inspecting anomalous difference electron density maps using Coot.^26^ Ligand positions were restrained to facilitate geometry optimization. Refinement statistics are reported in Table S1 of the SI. The structures were deposited in the PDB under the accession codes 30JS, 30JU, 30JX and 30JY.

### EPR Study of Reduction in Water

Cs_2_[V^V^_2_O_4_(lact)_2_]·2H_2_O was dissolved in an aqueous solution with phosphate-buffered saline (PBS) at pH 7.4 to obtain a V concentration of 4 mM. To this solution, an appropriate amount of ascorbate (Asc) or reduced glutathione (GSH) was added at room temperature to have a V-to-Asc/GSH ratio of 1:10. EPR spectroscopy was employed to monitor the reduction of V^V^ to V^IV^ as a function of time. The total V^IV^ concentration formed after the reduction was determined from the intensity of the *M*_I_ = −1/2 transition in the isotropic spectrum. The paramagnetic V^IV^O products formed after the reduction were identified by recording anisotropic EPR spectra at 100 K on frozen solutions of the systems [V^V^_2_O_4_(lact)_2_]^2–^/Asc and [V^V^_2_O_4_(lact)_2_]^2–^/GSH with a molar ratio 1:10.

EPR spectra were recorded with an X-band (9.15 GHz) Varian E-9 spectrometer equipped with a variable temperature unit using the following instrumental settings: microwave frequency: 9.15 GHz; microwave power: 20 mW; time constant: 0.5 s; modulation frequency: 100 kHz; modulation amplitude: 0.4 mT.

### EPR Study of ROS Production

The capability to generate hydroxyl radicals (^•^OH) by lact and [V^V^_2_O_4_(lact)_2_]^2–^ was evaluated in the absence or presence of reductants (Asc or GSH). For comparison, systems with only V^IV^O^2+^, V^V^O_2_^+^ and [V^IV^O­(acac)_2_], where acac is acetylacetonate, were studied. Moreover, the Fe^2+^-containing system, taken as a reference, was tested. In all the systems, H_2_O_2_ was added to recreate the conditions of the organism that led to Fenton or Fenton-like reactions. The formation of ^•^OH was detected using DMPO (5,5-dimethyl-1-pyrroline *N*-oxide) as a spin-trapping agent (Figure S1) and by monitoring the intensity of the EPR signal of the paramagnetic adduct DMPO–OH. The [V^V^_2_O_4_(lact)_2_]^2–^ concentration was 2.5 × 10^–5^ M, the H_2_O_2_ concentration 1.0 × 10^–4^ M and the DMPO concentration 2.925 × 10^–3^ M. The molar ratio between the reductant and V^V^ was fixed at 5:1. The solutions containing V^IV^O^2+^, V^V^O_2_^+^, or Fe^2+^ were studied at the pH obtained upon dissolution of the metal salts (pH *ca*. 3) to avoid hydroxide precipitation and the complexation of the ions by OH^–^ ligands, the ones present at physiological pH (7.4).

The results are reported as percentages relative to the EPR signal intensity of a reference solution containing only Fe^2+^ (taken as 100%), and each value represents the mean of three independent measurements.

EPR spectra were recorded at room temperature in aqueous solutions with an X-band (9.15 GHz) Varian E-9 spectrometer using the following instrumental settings: microwave frequency: 9.15 GHz; microwave power: 20 mW; time constant: 0.25 s; modulation frequency: 100 kHz; modulation amplitude: 0.4 mT.

### Cell Culture and Cytotoxicity Assay

Immortalized human keratinocytes (HaCaT; Innoprot, Biscay, Spain), murine fibroblasts (BALB/c-3T3; ATCC, Manassas, VA, USA, used as additional noncancer cell control), human cervical cancer cells (HeLa; ATCC, Manassas, VA, USA), and human prostate cancer cells (PC-3; ATCC, Manassas, VA, USA) were cultured at 37 °C in a humidified atmosphere containing 5% CO_2_. HaCaT, BALB/c-3T3, and HeLa cells were cultured in Dulbecco’s Modified Eagle Medium (DMEM; formulation could be found in https://www.thermofisher.com/at/en/home/technical-resources/media-formulation.8.html), while PC-3 cells were grown in RPMI-1640 medium (formulation could be found in https://www.thermofisher.com/at/en/home/technical-resources/media-formulation.114.html). Culture media were supplemented with 10% fetal bovine serum, 2 mM l-glutamine, and a standard antibiotic mixture.

Cell viability following treatment with Cs_2_[V^V^_2_O_4_(mal)_2_]·2H_2_O, Cs_2_[V^V^_2_O_4_(lact)_2_]·2H_2_O, or cisplatin was determined using the MTT (3-(4,5-dimethylthiazol-2-yl)-2,5-diphenyltetrazolium bromide) assay.^27^ The cells were seeded in 96-well plates at a density of 2 × 10^3^ cells per well, except for BALB/c-3T3 cells, which were plated at 3 × 10^3^ cells per well. After 24 h, cells were incubated with increasing concentrations (1–100 μM) of each compound for 48 h. At the end of the incubation, cell viability was measured by means of the MTT colorimetric assay, according to previously described procedures.^27^ Cell survival was expressed as the percentage of viable cells grown in the presence of VCs compared to the controls, represented by untreated cells and cells grown with identical volumes of H_2_O. Each sample was tested in three independent analyses, each carried out in triplicate.

### Determination of Intracellular ROS Levels

Intracellular reactive oxygen species (ROS) levels were determined using the fluorescent probe 2′,7′-dichlorodihydrofluorescein diacetate (DCFDA; Sigma-Aldrich), following the procedure previously described,^28^ with some modifications. Briefly, after seeding, cells were treated with IC_50_ concentrations of Cs_2_[V^V^_2_O_4_(mal)_2_]·2H_2_O or Cs_2_[V^V^_2_O_4_(lact)_2_]·2H_2_O for different times (1, 6, and 24 h) and subsequently incubated with the cell-permeable DCFDA probe. Following intracellular oxidation by ROS, the resulting fluorescence was measured using a PerkinElmer LS50 spectrofluorometer (excitation wavelength: 488 nm; emission wavelength: 525 nm; scanning speed: 300 nm min^–1^; slit width: 5 nm for both excitation and emission). ROS production was expressed as the percentage of DCF fluorescence intensity relative to untreated control cells. Results are presented as the mean of three independent experiments, each performed in triplicate. Statistical significance was evaluated using Student’s *t*-test.

## Results and Discussion

### **S**olution Behavior of Cs_2_[V^V^_2_O_4_(lact)_2_]·2H_2_O in the Absence and Presence of Lysozyme: ^51^V NMR Studies

The behavior of Cs_2_[V^V^_2_O_4_(lact)_2_]·2H_2_O in solution was first investigated by collecting UV–vis absorption spectra of the complex in the absence and presence of lysozyme in 1.1 M sodium chloride, 0.1 M sodium acetate, pH 4.0 (data not shown). The spectra do not significantly change over time, both with or without lysozyme. To analyze in more detail the behavior of Cs_2_[V^V^_2_O_4_(lact)_2_]·2H_2_O in solution, ^51^V NMR spectra of the compound (concentration = 5 mM) were collected under the conditions used to obtain the structures of its adduct with lysozyme (see below): (i) 1.1 M sodium chloride, 0.1 M sodium acetate, pH 4.0, 10% D_2_O; (ii) 20% ethylene glycol, 0.1 M sodium acetate, pH 4.0, 0.6 M sodium nitrate, 10% D_2_O; (iii) 0.8 M succinic acid, pH 7.0, 10% D_2_O; (iv) 2.0 M sodium formate, 0.1 M HEPES, pH 7.5, 10% D_2_O. Measurements were performed in the absence and presence of the protein (concentration = 13 mg/mL, 0.9 mM) after 1 h and 7 days of incubation at RT. Spectra collected in D_2_O were used as a reference (Figure 2). Data were compared with those already reported in the literature for VCs with α-hydroxycarboxylates, including malate.^6,7,9,29–34^

**Figure 2:**
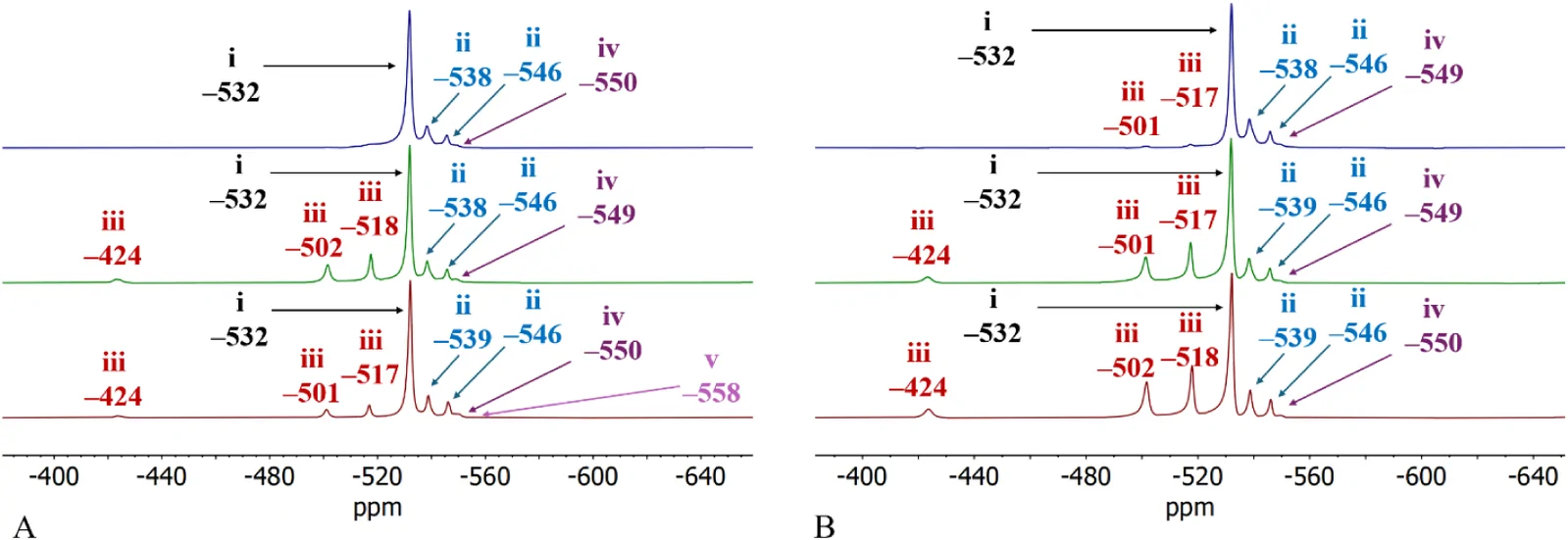
^51^V NMR spectra measured at RT after A) 1 h and B) 7 d of incubation of Cs_2_[V^V^_2_O_4_(lact)_2_]·2H_2_O (5 mM) dissolved in D_2_O (red lines); in 1.1 M sodium chloride, 0.1 M sodium acetate, pH 4.0, and 10% D_2_O (green lines); and in 1.1 M sodium chloride, 0.1 M sodium acetate, pH 4.0, and 10% D_2_O in the presence of lysozyme (blue lines). i indicates [V^V^_2_O_4_(lact)_2_]^2–^, ii [V^V^_3_O_7_(lact)_2_]^3–^, iii V_10_, iv [V^V^O_2_]^+^, and v [H_2_V^V^O_4_]^−^.

The spectrum of Cs_2_[V^V^_2_O_4_(lact)_2_]·2H_2_O collected at RT after 1 h of incubation in D_2_O reveals the presence of [V^V^_2_O_4_(lact)_2_]^2–^ (signal at ∼−532 ppm), [V^V^_3_O_7_(lact)_2_]^3–^ (signals at ∼−539 ppm, −546 ppm), [V^V^_10_O_28_]^6–^ (V_10_) (signals at ∼−424 ppm, −5012 ppm, −517 ppm), [V^V^O_2_]^+^ (∼−550 ppm), and [H_2_V^V^O_4_]^−^ (∼−558 ppm) (Figure 2). The formation of the trimetallic species like [V^V^_3_O_7_(lact)_2_]^3–^ in solution has not been found in the case of the similar complex Cs_2_[V^V^_2_O_4_(mal)_2_]·2H_2_O,^19^ but it has been previously observed in systems containing lactate, mandelate, and H_2_V^V^O_4_^–^.^6,7,29^ After 7 d of incubation, the same species are detected except for [H_2_V^V^O_4_]^−^. The quantitative analysis indicates that the percentage of decavanadate significantly increases over time: it is ∼12% after 1 h of incubation and ∼36% after 7 days (Figure 2, Table 1).

**Table 1:** Chemical Shifts in^51^V NMR Spectra Measured in Triplicate at RT after 1 h and 7 d of Incubation of Cs_2_[V^V^_2_O_4_(lact)_2_]·2H_2_O (5 mM) Dissolved in (i) D_2_O; (ii) 1.1 M Sodium Chloride, 0.1 M Sodium Acetate, pH 4.0, and 10% D_2_O; (iii) 1.1 M Sodium Chloride, 0.1 M Sodium Acetate, pH 4.0, and 10% D_2_O in the Presence of Lysozyme^a^

^51^V chemical shift (ppm)					Integration (%)			Sum			
Sample 1	Sample 2	Sample 3	Species	References	Sample 1	Sample 2	Sample 3	Sample 1	Sample 2	Sample 3	Average ± SD (%)
5 mM Cs_2_[V^V^_2_O_4_(lact)_2_]·2H_2_O in D_2_O (after 1 h at RT)											
–425	–424	–424	V_10_	^29,31^	0.7	1.3	1.3	13.1	10.8	11.8	11.9 ± 1.2
–501	–501	–501	V_10_ (or [V^V^O_2_(lact)(H_2_O)]^−^)	^6,29–33^	5.1	4.2	4.6				
–517	–517	–517	V_10_ (or [V^V^O_2_(lact)(OH)]^2–^)	^6,29–33^	7.3	5.3	5.9				
–532	–532	–532	[V^V^_2_O_4_(lact)_2_]^2–^	^6,29–33^	65.2	65.2	67.1	65.2	65.2	67.1	65.8 ± 1.1
–539	–539	–539	[V^V^_3_O_7_(lact)_2_]^3–^ (or [V^V^_2_O_5_(lact)]^2–^)	^6,29–33^	11.7	12.9	11.6	19.5	20.5	19.1	19.7 ± 0.7
–546	–546	–546	[V^V^_3_O_7_(lact)_2_]^3–^ (or [V^V^_2_O_5_(lact)]^2–^)	^6,29–33^	7.8	7.6	7.5				
–550	–550	–550	[V^V^O_2_]^+^	^36^	1.5	3.1	1.6	1.5	3.1	1.6	2.1 ± 0.9
–558	–558	–558	[H_2_V^V^O_4_]^−^	^6,29,36,37^	0.7	0.4	0.4	0.7	0.4	0.4	0.5 ± 0.2
5 mM Cs_2_[V^V^_2_O_4_(lact)_2_]·2H_2_O in 0.1 M NaOAc, pH 4.0, 1.1 M NaCl, 10% D_2_O (after 1 h at RT)											
–424	–424	–424	V_10_	^29,31^	2.5	1.3	1.2	25.2	27.9	24.0	25.7 ± 2.0
–501	–502	–502	V_10_ (or [V^V^O_2_(lact)(H_2_O)]^−^)	^6,29–33^	10.0	10.3	9.3				
–518	–518	–517	V_10_ (or [V^V^O_2_(lact)(OH)]^2–^)	^6,29–33^	12.7	16.3	13.5				
–532	–532	–532	[V^V^_2_O_4_(lact)_2_]^2–^	^6,29–33^	55.9	58.1	60.1	55.9	58.1	60.1	58.0 ±2.1
–538	–538	–538	[V^V^_3_O_7_(lact)_2_]^3–^ (or [V^V^_2_O_5_(lact)]^2–^)	^6,29–33^	12.1	8.7	9.9	17.4	12.7	14.8	15.0 ± 2.4
–546	–546	–546	[V^V^_3_O_7_(lact)_2_]^3–^ (or [V^V^_2_O_5_(lact)]^2–^)	^6,29–33^	5.3	4.0	4.9				
–549	–549	–549	[V^V^O_2_]^+^	^36^	1.5	1.3	1.1	1.5	1.3	1.1	1.3 ± 0.2
5 mM Cs_2_[V^V^_2_O_4_(lact)_2_]·2H_2_O + 13 mg/mL Lysozyme in 0.1 M NaOAc, pH 4.0, 1.1 M NaCl, 10% D_2_O (after 1 h at RT)											
–532	–532	–532	[V^V^_2_O_4_(lact)_2_]^2–^	^6,29–33^	76.4	76.1	76.6	76.4	76.1	76.6	76.4 ± 0.3
–538	–538	–538	[V^V^_3_O_7_(lact)_2_]^3–^ (or [V^V^_2_O_5_(lact)]^2–^)	^6,29–33^	15.1	15.8	15.5	22.1	22.6	21.7	22.1 ± 0.5
–546	–546	–546	[V^V^_3_O_7_(lact)_2_]^3–^ (or [V^V^_2_O_5_(lact)]^2–^)	^6,29–33^	7.0	6.8	6.2				
–550	–549	–550	[V^V^O_2_]^+^	^36^	1.5	1.3	1.7	1.5	1.3	1.7	1.5 ± 0.2
5 mM Cs_2_[V^V^_2_O_4_(lact)_2_]·2H_2_O in D_2_O (after 7 d at RT)											
–424	–425	–424	V_10_	^29,31^	4.2	2.9	3.3	38.4	34.7	34.6	35.9 ± 2.2
–502	–501	–502	V_10_ (or [V^V^O_2_(lact)(H_2_O)]^−^)	^6,29–33^	15.4	13.5	13.8				
–518	–517	–518	V_10_ (or [V^V^O_2_(lact)(OH)]^2–^)	^6,29–33^	18.8	18.3	17.5				
–532	–532	–532	[V^V^_2_O_4_(lact)_2_]^2–^	^6,29–33^	47.7	48.8	48.1	47.7	48.8	48.1	48.2 ± 0.6
–539	–539	–539	[V^V^_3_O_7_(lact)_2_]^3–^ (or [V^V^_2_O_5_(lact)]^2–^)	^6,29–33^	8.3	9.8	10.5	13.1	14.9	16.2	14.7 ±1.6
–546	–546	–546	[V^V^_3_O_7_(lact)_2_]^3–^ (or [V^V^_2_O_5_(lact)]^2–^)	^6,29–33^	4.8	5.1	5.7				
–550	–550	–550	[V^V^O_2_]^+^	^36^	0.8	1.6	1.1	0.8	1.6	1.1	1.2 ± 0.4
5 mM Cs_2_[V^V^_2_O_4_(lact)_2_]·2H_2_O in 0.1 M NaOAc, pH 4.0, 1.1 M NaCl, 10% D_2_O (after 7 d at RT)											
–424	–424	–424	V_10_	^29,31^	3.4	3.2	3.0	29.3	32.6	31.0	31.0 ± 1.7
–502	–501	–501	V_10_ (or [V^V^O_2_(lact)(H_2_O)]^−^)	^6,29–33^	11.5	13.3	12.1				
–518	–517	–517	V_10_ (or [V^V^O_2_(lact)(OH)]^2–^)	^6,29–33^	14.4	16.1	15.9				
–532	–532	–532	[V^V^_2_O_4_(lact)_2_]^2–^	^6,29–33^	50.9	52.5	53.3	50.9	52.5	53.3	52.2 ± 1.2
–539	–539	–539	[V^V^_3_O_7_(lact)_2_]^3–^ (or [V^V^_2_O_5_(lact)]^2–^)	^6,29–33^	12.4	9.5	10.0	18.2	14.0	14.6	15.6 ± 2.3
–546	–546	–546	[V^V^_3_O_7_(lact)_2_]^3–^ (or [V^V^_2_O_5_(lact)]^2–^)	^6,29–33^	5.8	4.5	4.6				
–549	–549	–549	[V^V^O_2_]^+^	^36^	1.6	0.9	1.1	1.6	0.9	1.1	1.2 ± 0.4
5 mM Cs_2_[V^V^_2_O_4_(lact)_2_]·2H_2_O + 13 mg/mL Lysozyme in 0.1 M NaOAc, pH 4.0, 1.1 M NaCl, 10% D_2_O (after 7 d at RT)											
–501	–501	–501	V_10_ (or [V^V^O_2_(lact)(H_2_O)]^−^)	^6,29–33^	0.9	3.4	1.3	3.0	9.6	5.4	6.0 ± 3.3
–517	–517	–517	V_10_ (or [V^V^O_2_(lact)(OH)]^2–^)	^6,29–33^	2.1	6.2	4.1				
–532	–532	–532	[V^V^_2_O_4_(lact)_2_]^2–^	^6,29–33^	67.2	69.4	72.9	67.2	69.4	72.9	69.8 ± 2.9
–539	–538	–538	[V^V^_3_O_7_(lact)_2_]^3–^ (or [V^V^_2_O_5_(lact)]^2–^)	^6,29–33^	19.2	13.9	14.7	27.7	20.1	20.9	22.9 ± 4.2
–546	–546	–546	[V^V^_3_O_7_(lact)_2_]^3–^ (or [V^V^_2_O_5_(lact)]^2–^)	^6,29–33^	8.5	6.2	6.2				
–549	–549	–549	[V^V^O_2_]^+^	^36^	2.1	0.9	0.8	2.1	0.9	0.8	1.3 ± 0.7

a The relative content of species was calculated based on the integration of ^51^V signals. Signals were assigned based on literature data .

The spectra collected in 1.1 M sodium chloride, 0.1 M sodium acetate, pH 4.0, and 10% D_2_O show the presence of [V^V^_2_O_4_(lact)_2_]^2–^, [V^V^_3_O_7_(lact)_2_]^3–^, V_10_, and [V^V^O_2_]^+^ after both incubation times. Also, in this case, the integration analysis shows that the amount of decavanadate increases over time (Figure 2, Table 1). In contrast, in the presence of the protein, only [V^V^_2_O_4_(lact)_2_]^2–^, [V^V^_3_O_7_(lact)_2_]^3–^, and [V^V^O_2_]^+^ are detected after 1 h of incubation, while a small amount (∼6%) of V_10_ is observed after 7 days (Figure 2, Table 1).

The ^51^V NMR spectrum of Cs_2_[V^V^_2_O_4_(lact)_2_]·2H_2_O was also collected at RT after 1 h of incubation in 20% ethylene glycol (EG), 0.1 M sodium acetate, pH 4.0, 0.6 M sodium nitrate, and 10% D_2_O. The results indicate the presence of [V^V^_2_O_4_(lact)_2_]^2–^ (signal at ∼−532 ppm), [V^V^_3_O_7_(lact)_2_]^3–^ (signals at ∼−538 ppm, −546 ppm), V_10_ (signals at ∼−425 ppm, −502 ppm, −520 ppm–522019ppm), and a dinuclear oxidovanadium­(V) complex with EG as a ligand, [V^V^_2_O_4_(EG)_2_]^2–^ (signal at ∼−523 ppm)^35^ (Figure S2). After 7 d of incubation, the same species are detected, with the addition of [V^V^O_2_(lact)­(H_2_O)]^−^ (signal at ∼−509 ppm) and [V^V^O_2_(lact)­(OH)]^2–^ (signal at ∼−520 ppm) (Figure S2, Table S2). In the presence of lysozyme, only [V^V^_2_O_4_(lact)_2_]^2–^, [V^V^_3_O_7_(lact)_2_]^3–^, [V^V^_2_O_4_(EG)_2_]^2–^, and [V^V^O_2_(lact)­(OH)]^2–^ are observed (Figure S2, Table S2).

Finally, the spectra registered in 0.8 M succinic acid, pH 7.0, and 10% D_2_O both in the absence and presence of lysozyme reveal the presence of [V^V^_2_O_4_(lact)_2_]^2–^ (signal at ∼−532 ppm), [V^V^_3_O_7_(lact)_2_]^3–^ (signals at ∼−540 ppm, −549 ppm), [H_2_V^V^O_4_]^−^ (∼−557 ppm), [H_2_V^V^_2_O_7_]^2–^ (∼−570 ppm), [V^V^_4_O_12_]^4–^ (∼−574 ppm), and [V^V^_5_O_15_]^5–^ (∼−582 ppm) (Figure S3, Table S3). Similar results are obtained in 2.0 M sodium formate, 0.1 M HEPES, pH 7.5, and 10% D_2_O. The only significant difference is a signal attributed to [V^V^_2_O_4_(lact)_2_]^2–^, which in the latter condition is observed after 7 d of incubation (Figure S4, Table S4).

### Solution Behavior of Cs_2_[V^V^_2_O_4_(lact)_2_]·2H_2_O in the Absence and Presence of Lysozyme: Mass Spectrometry Data

The ESI mass spectrum of Cs_2_[V^V^_2_O_4_(lact)_2_]·2H_2_O in water in negative ion mode shows a series of singly and doubly charged species. The singly charged series includes protonated and unprotonated vanadates [V^V^O_3_]^−^, [H_2_V^V^O_4_]^−^, [HV^V^_2_O_6_]^−^, [V^V^_3_O_8_]^−^, and [HV^V^_4_O_11_]^−^, the monoanionic lact^2–^ ligand, and various vanadium ions with one or two metal centers: [V^V^O_2_(lact)]^−^, [V^V^O­(lact)_2_]^−^, [V^V^_2_O_4_(lact) – H^+^]^−^, [V^V^_2_O_4_(lact)­(OH)]^−^, [V^V^_2_O_5_(lact)­(H_2_O)^2–^ + Na^+^]^−^, [V^V^_2_O_4_(lact)_2_^2–^ + H^+^]^−^, and [V^V^_2_O_4_(lact)_2_^2–^ + Cs^+^]^−^. The only doubly charged species that were identified are [V^V^_10_O_26_]^2–^ and [H_4_V^V^_10_O_28_]^2–^ (Figure S5A, Table 2). The negative ion mode spectrum of Cs_2_[V^V^_2_O_4_(lact)_2_]·2H_2_O in a mixture of acetonitrile/methanol + 1% H_2_O is shown in Figure S5B. In this spectrum, most of the species revealed in water can be observed (Table 2) except for [V^V^_2_O_4_(lact) – H^+^]^−^, [V^V^_3_O_8_]^−^, [V^V^_2_O_4_(lact)_2_^2–^ + Cs^+^]^−^, and decavanadates.

**Table 2:** Experimental and Calculated *m*/*z* Values for Cs_2_[V^V^_2_O_4_(lact)_2_]·2H_2_O Dissolved in H_2_O and in ACN/MeOH + 1% H_2_O, Recorded in Negative Ion Mode Using ESI-MS

**Observed species**	**Experimental** *m*/*z*	**Simulated** *m*/*z*
**In H_2_O**		
[lact^2–^ + H^+^]^−^	89.02	89.02
[V^V^O_3_]^−^	98.93	98.93
[H_2_V^V^O_4_]^−^	116.94	116.94
[V^V^O_2_(lact)]^−^	170.95	170.95
	171.95	171.95
[HV^V^_2_O_6_]^−^	198.87	198.87
[V^V^O(lact)_2_]^−^	242.97	242.97
	243.97	243.97
[V^V^_2_O_4_(lact) – H^+^]^−^	252.88	252.88
	253.88	253.88
[V^V^_2_O_4_(lact)(OH)]^−^	270.89	270.89
	271.89	271.89
[V^V^_3_O_8_]^−^	280.79	280.79
[V^V^_2_O_5_(lact)(H_2_O)^2–^ + Na^+^]^−^	310.88	310.88
	311.89	311.88
	312.89	312.88
[V^V^_2_O_4_(lact)_2_^2–^ + H^+^]^−^	342.91	342.91
	343.91	343.91
	344.91	344.91
[HV^V^_4_O_11_]^−^	380.73	380.73
[V^V^_10_O_26_]^2–^	462.16	462.16
	462.65	462.65
	463.16	463.16
	463.66	463.66
	464.16	464.16
	464.65	464.65
[V^V^_2_O_4_(lact)_2_^2–^ + Cs^+^]^−^	474.81	474.81
	475.81	475.81
[H_4_V^V^_10_O_28_]^2–^	480.17	480.17
	480.67	480.67
	481.17	481.17
	481.67	481.67
**In** **ACN/MeOH + 1% H_2_O**		
[lact^2–^ + H^+^]^−^	89.02	89.02
[V^V^O_3_]^−^	98.93	98.93
[H_2_V^V^O_4_]^−^	116.94	116.94
[V^V^O_2_(lact)]^−^	170.95	170.95
	171.95	171.95
[HV^V^_2_O_6_]^−^	198.87	198.87
[V^V^O(lact)_2_]^−^	242.97	242.97
	243.97	243.97
[V^V^_2_O_4_(lact)(OH)]^−^	270.89	270.89
	271.89	271.89
[V^V^_2_O_5_(lact)(H_2_O)^2–^ + Na^+^]^−^	310.88	310.88
	311.89	311.88
[V^V^_2_O_4_(lact)_2_^2–^ + H^+^]^−^	342.91	342.91
	343.91	343.91
	344.91	344.91
[HV^V^_4_O_11_]^−^	380.73	380.73

The positive ion mode ESI-MS spectrum of lysozyme in the presence of Cs_2_[V^V^_2_O_4_(lact)_2_]·2H_2_O (VC/lysozyme molar ratio = 1:1) in H_2_O shows two intense peaks at 14304.9 Da and 14648.7 Da, respectively. The former peak belongs to the metal-free protein, while the latter is attributed to the [V^V^_2_O_4_(lact)_2_^2–^]+2H^+^]–lysozyme adduct (Figure 3). Upon 1:100 dilution in acetonitrile/H_2_O 9/1 + 1% formic acid (FA), the spectrum shows the peaks of the metal-free protein and of an adduct of the protein with [V^V^O­(lact)_2_]^−^.

**Figure 3:**
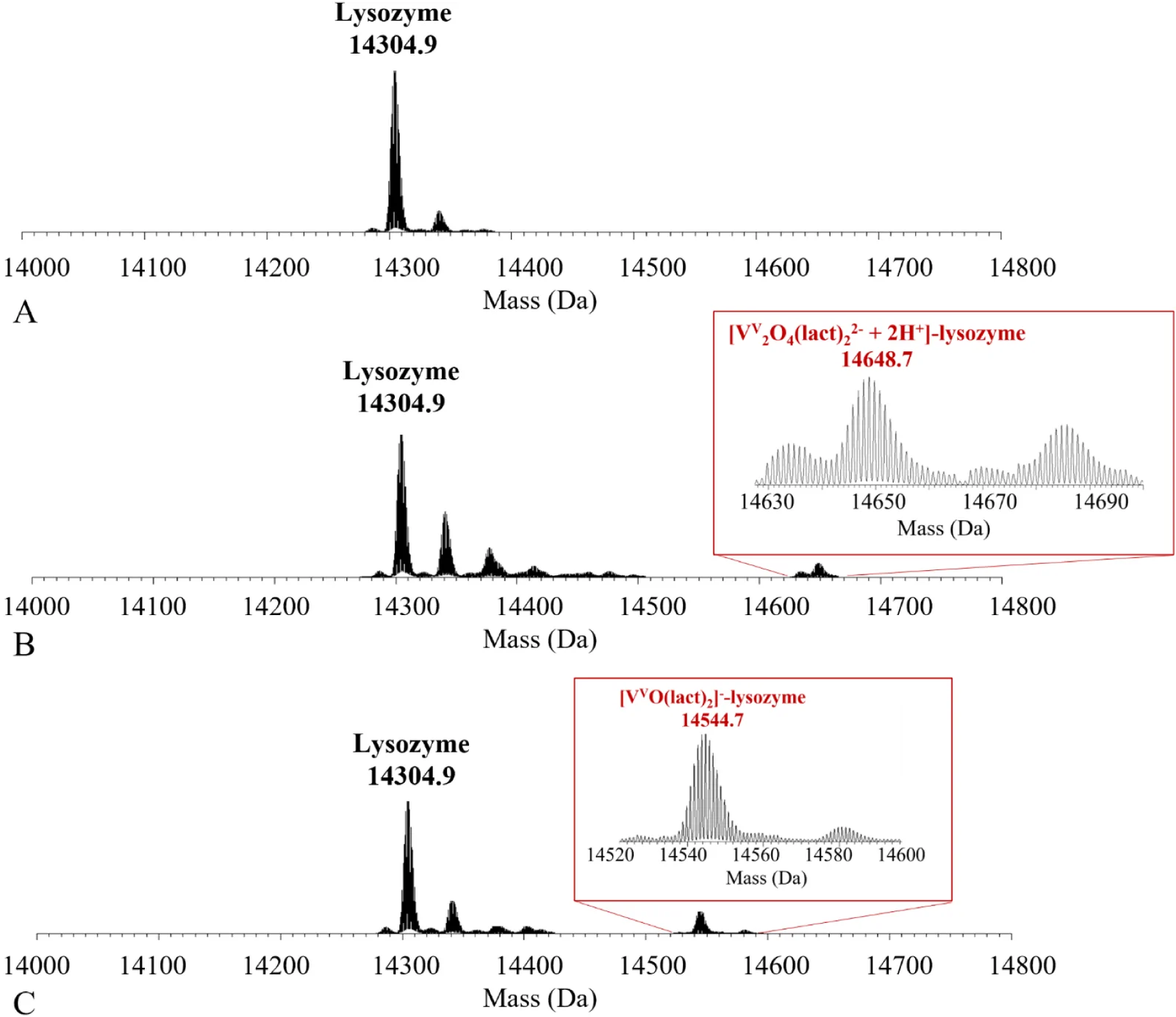
Deconvoluted ESI-MS spectra of the systems: A) 1:100 diluted lysozyme (concentration = 13 mg/mL, 0.9 mM) in acetonitrile/H_2_O 9/1 + 1% FA; B) Cs_2_[V^V^_2_O_4_(lact)_2_]·2H_2_O/lysozyme in 1:1 molar ratio in H_2_O; C) Cs_2_[V^V^_2_O_4_(lact)_2_]·2H_2_O/lysozyme in a 1:1 molar ratio diluted 1:100 in acetonitrile/H_2_O 9/1 + 1% FA. Peaks attributed to the adduct are magnified in the insets.

### Effect of the Presence of Cs_2_[V^V^_2_O_4_(lact)_2_]·2H_2_O on the Secondary Structure of Lysozyme in Solution: Circular Dichroism Data

To analyze the effect of the presence of Cs_2_[V^V^_2_O_4_(lact)_2_]·2H_2_O on the secondary structure of lysozyme, circular dichroism (CD) spectra were collected in 0.01 M sodium acetate buffer, pH 4.0, at different protein-to-VC molar ratios (from 1:1 to 1:5) and after different incubation times (1 h, 48 h and 7 days). The CD spectra, reported in Figure S6, indicate that the presence of Cs_2_[V^V^_2_O_4_(lact)_2_]·2H_2_O does not significantly affect the secondary structure content of lysozyme.

### X-ray Structures of the Adducts Formed by Cs_2_[V^V^_2_O_4_(lact)_2_]·2H_2_O with Lysozyme

X-ray diffraction data were collected at 100 K on crystals of the adducts formed by lysozyme with Cs_2_[V^V^_2_O_4_(lact)_2_]·2H_2_O under the same experimental conditions used to study its in-solution behavior by ^51^V NMR. High-resolution crystal structures were solved at 1.20–1.49 Å resolution. The data collection and refinement statistics are reported in Table S1.

The overall structures of lysozyme in the adducts are very similar to each other and to the metal-free protein, with root-mean-square deviations between Cα atoms in the range of 0.15–0.47 Å (Figure 4).

**Figure 4:**
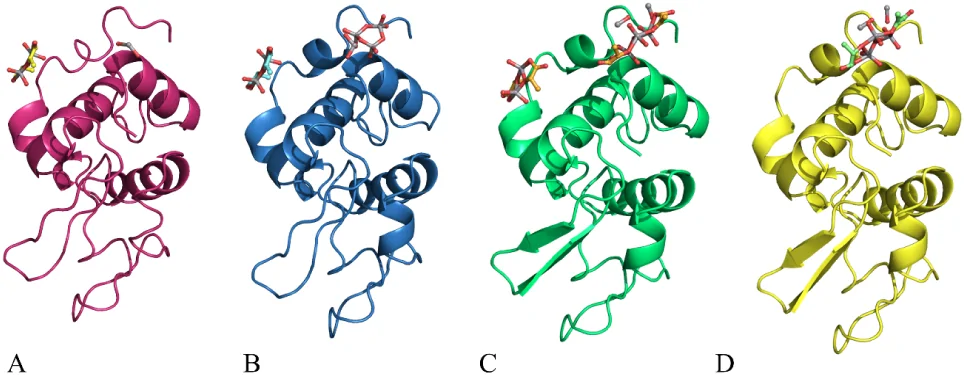
Overall structure of the adduct formed upon reaction of Cs_2_[V^V^_2_O_4_(lact)_2_]·2H_2_O with lysozyme from crystals grown in: A) 1.1 M sodium chloride, 0.1 M sodium acetate, pH 4.0 (structure **A**); B) 20% ethylene glycol, 0.1 M sodium acetate, pH 4.0, 0.6 M sodium nitrate (structure **B**); C) 0.8 M succinic acid, pH 7.0 (structure **C**); and D) 2.0 M sodium formate, 0.1 M HEPES, pH 7.5 (structure **D**). Vanadium is shown in gray.

Structure **A** (Figure 4A) corresponds to the lysozyme adduct with Cs_2_[V^V^_2_O_4_(lact)_2_]·2H_2_O formed in 1.1 M sodium chloride and 0.1 M sodium acetate at pH 4.0. The model refines to R-factor/R-free values of 0.174/0.201 at 1.20 Å resolution. In this structure, two V-containing fragments noncovalently interact with the protein: a VO_2_ group (occupancy = 0.70) and a [V^V^_2_O_4_(lact)_2_]^2–^ dianion (occupancy = 0.80). The VO_2_ group, assigned to [V^V^O_2_]^+^, is stabilized through hydrogen bonds with the main-chain N atoms of Cys6 and Glu7 and with water molecules (Figure 5A). [V^V^_2_O_4_(lact)_2_]^2–^ interacts with the NZ atom of Lys33, water molecules, and with the main-chain O atoms of Gly117 and Thr118, the main-chain N atom and side-chain OE1 atom of Gln121 from a symmetry-related molecule (Figure 5B). The species identified in the crystal structure were also observed in ^51^V NMR spectra collected under the same experimental conditions (Figure 2), which showed the presence of [V^V^_2_O_4_(lact)_2_]^2–^, [V^V^O_2_]^+^, and the trimetallic species [V^V^_3_O_7_(lact)_2_]^3–^. Geometrical details of the structure of [V^V^_2_O_4_(lact)_2_]^2–^ are compared with those reported for the Cambridge Structural Database in Table 3.

**Figure 5:**
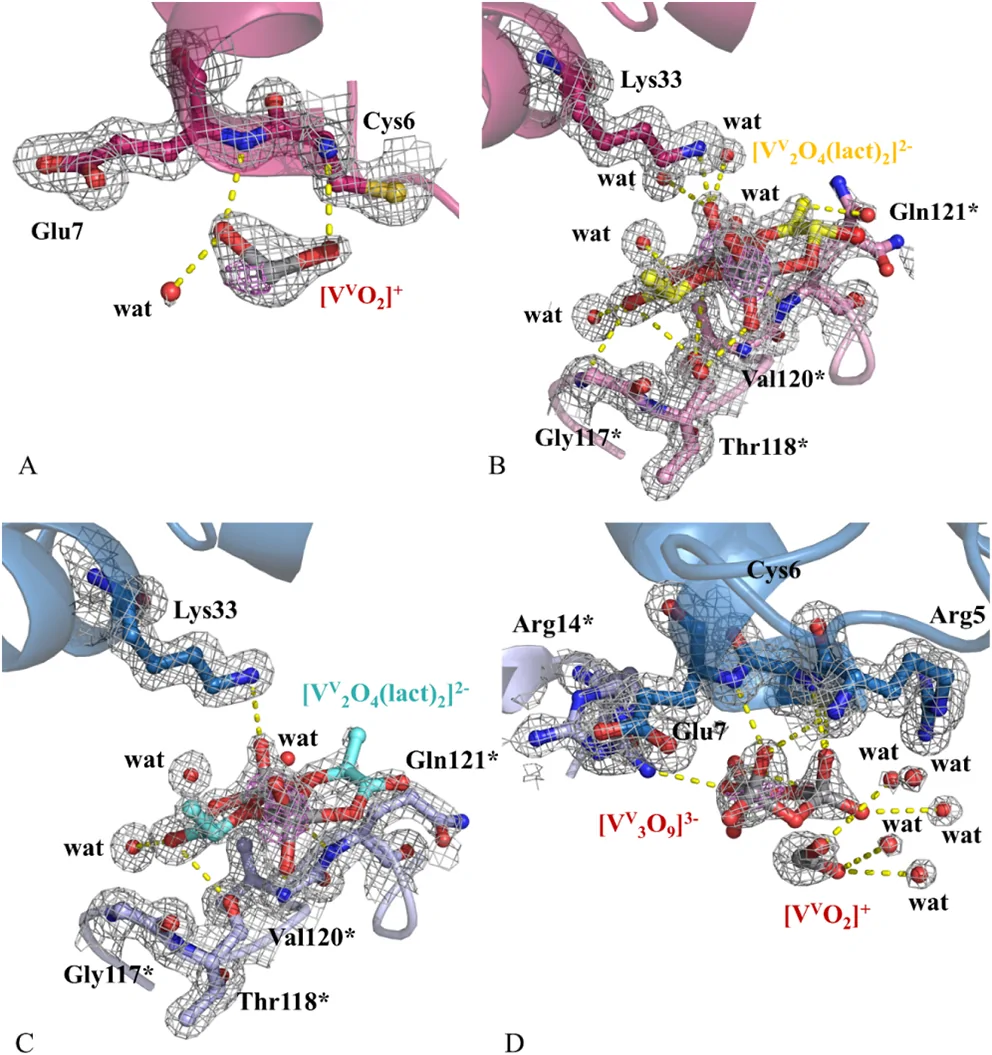
Vanadium binding sites in structures **A** and **B**. Binding sites of [V^V^O_2_]^+^ (panel A) and [V^V^_2_O_4_(lact)_2_]^2–^ in structures **A** and **B** (panels B–C), and of [V^V^_3_O_9_]^3–^ and [V^V^O_2_]^+^ in structure **B** (D). 2Fo–Fc electron density maps are contoured at the 1.0 σ level and depicted in gray. The anomalous difference electron density maps are reported in violet at the 3.0 σ.

**Table 3:** Geometrical Details of the Structures of Cs_2_[V^V^_2_O_4_(lact)_2_]·2H_2_O Compared with Those Reported for the Cambridge Crystallographic Data Centre (CCDC Code: QAVCOF).^20^

	Cs_2_[V^V^_2_O_4_(lact)_2_]·2H_2_O CCDC code: QAVCOF^20^		Cs_2_[V^V^_2_O_4_(lact)_2_]·2H_2_O/lysozyme Structure **A**		Cs_2_[V^V^_2_O_4_(lact)_2_]·2H_2_O/lysozyme Structure **B**		Cs_2_[V^V^_2_O_4_(lact)_2_]·2H_2_O/lysozyme Structure **C**	
***Bond***	**Length (Å)**		**Length (Å)**		**Length (Å)**		**Length (Å)**	
V–O(oxido)	1.617	1.635	1.69	1.79	1.70	1.79	1.71	1.81
	1.599	1.605	1.79	1.71	1.80	1.73	1.81	1.74
V–O(carb)	1.974	1.979	2.32	2.09	2.34	2.12	2.33	2.14
V–O(alc)	1.998	2.004	2.45	1.98	2.48	1.99	2.48	2.01
V–O′(alc)	1.949	1.965	2.02	1.94	2.03	1.96	2.06	1.94

***Angle***	**Value (°)**		**Value (°)**		**Value (°)**		**Value (°)**	
O(oxido)–V–O(oxido)	107.9	108.8	129.33	109.34	127.24	106.74	125.46	107.97
O(oxido)–V–O(alc)	121.2	130.9	96.65	133.92	99.06	133.60	117.88	116.37
	124.5	126.8	121.07	129.54	123.30	129.74	128.39	123.55
O(oxido)–V–O′(alc)	99.7	102.2	92.29	97.58	93.26	97.83	90.97	107.52
	99.7	101.0	89.39	94.18	92.29	94.69	87.44	95.76
O(alc)–V–O′(alc)	71.6	71.2	75.02	88.66	74.13	88.22	75.46	90.12
O(carb)–V–O(alc)	77.1	76.4	75.92	76.14	74.58	74.24	74.58	76.44
O(carb)–V–O′(alc)	148.2	147.6	149.19	164.55	147.57	162.35	148.31	161.20
V–O–V	108.8	108.8	90.28	103.12	90.57	104.41	87.84	99.09

Structure **B** was refined at 1.34 Å resolution using data collected from crystals grown in 20% ethylene glycol, 0.1 M sodium acetate at pH 4.0, and 0.6 M sodium nitrate (Figure 4B). Three V-containing species were identified: [V^V^_2_O_4_(lact)_2_]^2–^ (occupancy = 0.80) (Figure 5C), [V^V^O_2_]^+^ and a cyclic [V^V^_3_O_9_]^3–^ (occupancy = 0.50) (Figure 5D). These species noncovalently bind to the protein. [V^V^_2_O_4_(lact)_2_]^2–^ occupies the same position and forms the same interactions with protein residues observed in structure **A**. In contrast, the [V^V^O_2_]^+^ ion is not observed in the same position found in structure **A**. It forms hydrogen bonds only with solvent molecules (Figure 5D) and is an alternative to [V^V^_3_O_9_]^3–^, which interacts with the N atoms of Arg5, Cys6, and Glu7, and with several water molecules (Figure 5D). The crystallographic data are in good agreement with the speciation trends revealed by ^51^V NMR spectra collected under the same experimental conditions, which showed the presence of [V^V^_2_O_4_(lact)_2_]^2–^ and [V^V^O_2_]^+^ (Figure S2). However, ^51^V NMR spectra additionally revealed a dimeric species containing ethylene glycol as a ligand, [V^V^O_2_(lact)­(OH)]^2–^, and [V^V^_3_O_7_(lact)_2_]^3–^ (Figure S2), whereas crystallographic results exhibited the presence of the cyclic [V^V^_3_O_9_]^3–^, a species that has never been found before in an adduct with a protein.

In the structure **C** (1.49 Å resolution, 0.8 M succinic acid, pH 7.0) (Figure 4C), two [V^IV^O]^2+^ ions (occupancy = 0.40), one [V^V^_3_O_7_(lact)_2_]^3–^ anion (occupancy = 0.60) (Figure 6A), and one [V^V^_2_O_4_(lact)_2_]^2–^ (occupancy = 0.80) (Figure 6B) were identified. The species present in the crystal structure are observed also in the ^51^V NMR spectra in 0.8 M succinic acid at pH 7.0 (Figure S3). It must be observed that, however, ^51^V NMR data showed the presence of [H_2_V^V^O_4_]^−^, [H_2_V^V^_2_O_7_]^2–^, [V^V^_4_O_12_]^4–^, [V^V^_5_O_15_]^5–^ as well.

**Figure 6:**
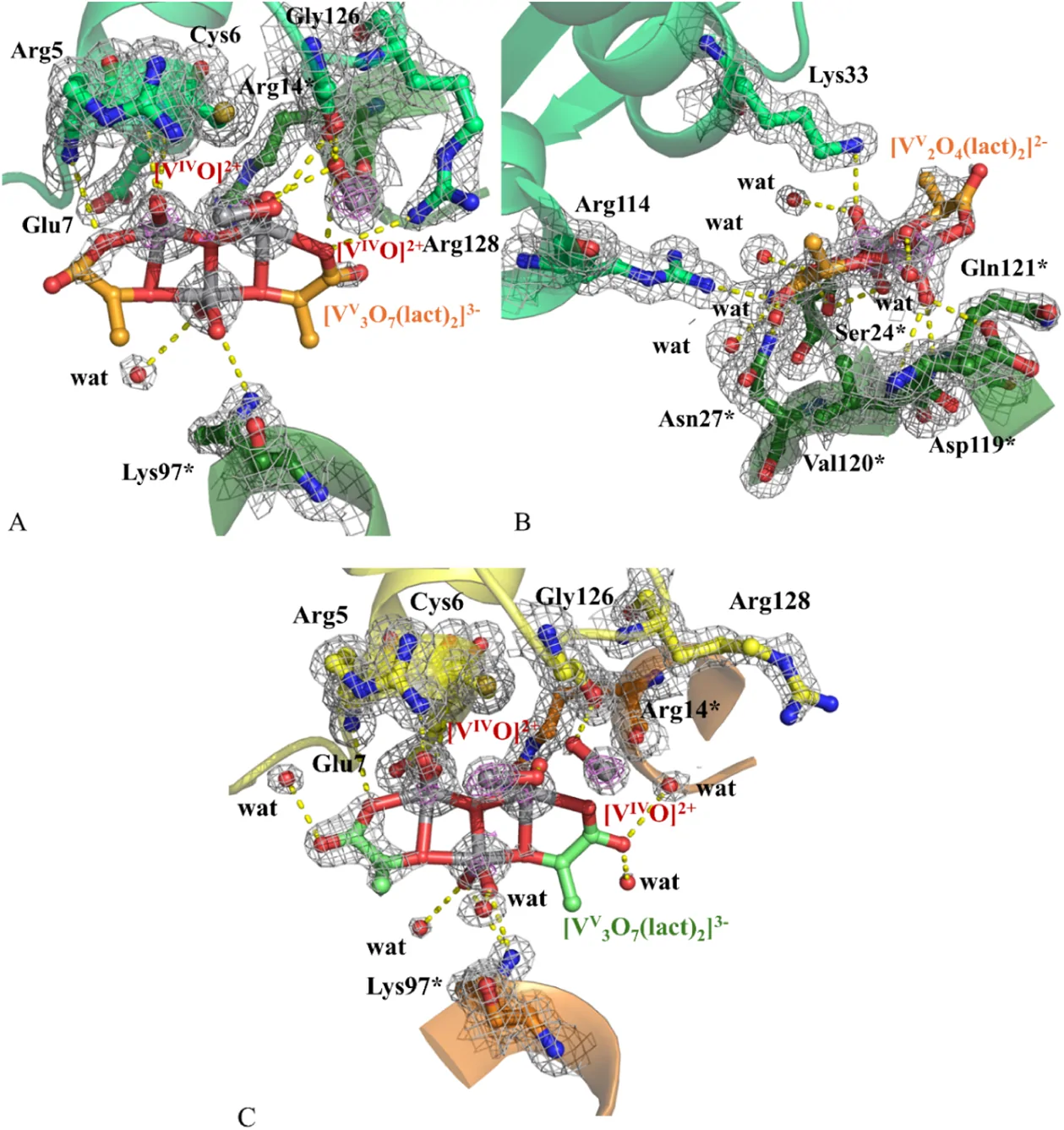
Vanadium binding sites in structures **C** and **D**. Binding sites of the two [V^IV^O]^2+^ ions and of [V^V^_3_O_7_(lact)_2_]^3–^instructure**C** ^–^–a are reported in panels A and C, respectively. The binding site of [V^V^_2_O_4_(lact)_2_]^2–^ in structure **C** is reported in panel B. The 2Fo–Fc electron density maps are contoured at the 1.0 σ level and depicted in gray. The anomalous difference electron density maps are reported at the 3.0 σ level in violet.

The structure of a protein adduct with [V^V^_3_O_7_(lact)_2_]^3–^ has never been reported before. It consists of three V centers, each coordinated by two terminal oxido ligands and three bridging oxygens, two of which belong to the lactate ligands (Figure 6A). The [V^V^_3_O_7_(lact)_2_]^3–^ anion forms hydrogen bonds with the N atoms of Arg5, Cys6, Glu7, the side chain of Arg128, a water molecule, the side chains of Arg14 and Lys97 from a symmetry-related molecule, as well as the oxygen of a neighboring [V^IV^O]^2+^ ion. [V^IV^O]^2+^, in turn, interacts with one water molecule and the O atom of Gly126 (Figure 6A). Additionally, both [V^V^_3_O_7_(lact)_2_]^3–^ and the interacting [V^IV^O]^2+^ ion are alternative to another [V^IV^O]^2+^ ion, which also interacts with the O of Gly126. Notably, the formation of adducts of [V^IV^O]^2+^ ions with lysozyme has been recently demonstrated in similar systems containing [V^V^_2_O_4_(mal)_2_]^2–^ instead of [V^V^_2_O_4_(lact)_2_]^2–^.^19^

The [V^V^_2_O_4_(lact)_2_]^2–^ ion is stabilized through an extended network of hydrogen bonds involving the side chains of Lys33 and Arg114, water molecules, the main chain of Val120 and Gln121, and the side chains of Ser24, Asn27, and Asp119 from a symmetry-related molecule (Figure 6B).

Structure **D** (Figure 4D) was solved using data collected at 1.23 Å from a crystal grown in 2.0 M sodium formate, 0.1 M HEPES, pH 7.5. As expected, based on ^51^V NMR data (Figure S4), this structure is very similar to structure **C** (Figure 6C). The only significant difference is the absence of [V^V^_2_O_4_(lact)_2_]^2–^.

In conclusion, the structural results reveal that Cs_2_[V^V^_2_O_4_(lact)_2_]·2H_2_O speciation and binding to lysozyme strongly depend on the experimental conditions, with the dinuclear species that predominates in acidic media, and trinuclear and mononuclear vanadates that are stabilized at neutral pH. This agrees well with the distribution of the species V^V^–lact as a function of pH calculated by Pettersson and coworkers by pH-potentiometry.^7^ This dynamic behavior underscores the high complexity of V coordination chemistry in the presence of proteins.

### Comparison with Literature Data Collected on Cs_2_[V^V^_2_O_4_(mal)_2_]·2H_2_O

Although the dinuclear complexes Cs_2_[V^V^_2_O_4_(mal)_2_]·2H_2_O and Cs_2_[V^V^_2_O_4_(lact)_2_]·2H_2_O share the same structure and ligands belonging to the α-hydroxycarboxylates that are very similar to each other, they exhibit different behaviors both in solution and in the solid state in the absence and presence of lysozyme.

The spectroscopic, spectrometric, and crystallographic data reveal that Cs_2_[V^V^_2_O_4_(mal)_2_]·2H_2_O readily transforms into a mixture of species containing two ([V^V^_2_O_4_(mal)_2_]^2–^ and [V^V^_2_O_5_(mal)]^2–^), or ten V atoms (V_10_), depending on pH, temperature, and the presence or absence of the protein.^19^ Crystallographic analysis indicates that lysozyme can stabilize several of these species, including [V^IV^O]^2+^, [V^V^_2_O_5_(mal)]^2–^, as well as decavanadate. The latter binds to the protein both noncovalently and covalently through the carboxylate groups of Glu7 and Glu7*. At physiological temperature, by contrast, the decavanadate anion does not form, and only an adduct with a [V^IV^O]^2+^ ion is detected, consistent with ^51^V NMR data.

In contrast, in aqueous solution, the dinuclear V^V^ complex with lactate gives [V^V^O_2_]^+^, [H_2_V^V^O_4_]^−^, [V^V^O_2_(lact)­(OH)]^2–^, and [V^V^O_2_(lact)­(H_2_O)]^−^, and transforms into a mixture of species containing two ([H_2_V^V^_2_O_7_]^2–^, [V^V^_2_O_4_(lact)_2_]^2–^), three ([V^V^_3_O_7_(lact)_2_]^3–^), four ([V^V^_4_O_12_]^4–^), five ([V^V^_5_O_15_]^5–^), and ten V atoms (V_10_). Across the different crystallization conditions, lysozyme binds [V^V^_2_O_4_(lact)_2_]^2–^, [V^IV^O]^2+^, [V^V^O_2_]^+^, [V^V^_3_O_9_]^3–^, and [V^V^_3_O_7_(lact)_2_]^3–^. The interaction between V-containing fragments and proteins is mainly governed by hydrogen bonds with surface residues, indicating that the lactate ligand stabilizes the adducts through noncovalent interactions. These results highlight the influence of the ligand on speciation and interaction with proteins in these systems, which could affect the biological properties of these complexes such as the transport in blood and in the cellular environment, the binding with receptors and, finally, the pharmacological activity.^38,39^

### Biological Activity

The cytotoxic activity of Cs_2_[V^V^_2_O_4_(mal)_2_]·2H_2_O and Cs_2_[V^V^_2_O_4_(lact)_2_]·2H_2_O was evaluated on two immortalized cell lines (HaCaT and BALB/c-3T3) and two cancer cell lines (HeLa and PC-3) by MTT assay after 48 h incubation. IC_50_ values, defined as the concentration (μM) able to induce 50% cell death, are reported in Table 4. Dose–response curves are reported in Figure S7. Both complexes exhibit cytotoxic activity in the low micromolar range on all tested cell lines. In particular, Cs_2_[V^V^_2_O_4_(mal)_2_]·2H_2_O and Cs_2_[V^V^_2_O_4_(lact)_2_]·2H_2_O display comparable activity but no selectivity toward cancer cell lines was observed, since the obtained IC_50_ values are similar for all the cell lines tested. These values are lower than those reported for inorganic vanadium salts, which show IC_50_ values within the range of 10–100 μM against Chinese Hamster Ovary Cells (CHO K1 cells), primary astrocytes, and DU 145 prostate cancer cells,^40–42^ and are comparable to those reported for other established V-based anticancer agents. For example, vanadocene dichloride (VDC) exhibits an IC_50_ value of 8.6 μM against HeLa cells after 24 h,^43^ while Metvan has been shown to exert very potent cytotoxicity in multiple solid tumor cell lines, including HeLa and PC-3, with IC_50_ values in the low micromolar to submicromolar range (approximately 0.5–2 μM after 24–48 h).^44^ Within this context, our dinuclear oxidovanadium­(V) complexes display cytotoxicity of the same order of magnitude as VDC, though they are less potent than Metvan, which remains one of the most active VC reported to date.

**Table 4:** IC_50_ Values (μM) Obtained for Cs_2_[V^V^_2_O_4_(mal)_2_]·2H_2_O and Cs_2_[V^V^_2_O_4_(lact)_2_]·2H_2_O on HaCaT, Balb/c-3T3, HeLa, and PC-3 Cells upon 48 h Incubation

**Cell line**	**Cs_2_[V^V^_2_O_4_(mal)_2_]·2H_2_O**	**Cs_2_[V^V^_2_O_4_(lact)_2_]·2H_2_O**	**Cisplatin**
HaCaT	8.0 ± 0.6	5.0 ± 0.3	6.6 ± 0.3^a^
BALB/c-3T3	6.0 ± 0.7	5.0 ± 0.6	240 ± 47^a^
HeLa	12.9 ± 0.6	17.0 ± 2.4	8.7 ± 2.4^b^
PC-3	5.0 ± 0.3	5.9 ± 0.3	29.9 ± 0.9^b^

a IC_50_ values of cisplatin are from ref 28.

b This work.

Cisplatin, used as a reference drug, exhibits variable activity, with stronger activity toward HeLa cells (IC_50_ value of 8.7 ± 2.4 μM) and significantly reduced cytotoxicity toward BALB/c-3T3 cells (240 ± 47 μM). Notably, both V^V^–lactate and V^V^–malate complexes show higher toxicity with respect to cisplatin on PC-3 cells, with IC_50_ values being 5- to 6-times lower (IC_50_ value of 5.9 ± 0.3 μM for the lactate complex and 5.0 ± 0.3 μM for the malate complex, compared to 29.9 ± 0.9 μM for cisplatin). Overall, these findings indicate that both vanadium complexes studied in this work possess broad cytotoxicity but lack selectivity under these experimental conditions.

### Reduction of [V^V^_2_O_4_(lact)_2_]^2–^ in the Presence of Cellular Reducing Agents

Complexes of labile metal ions are prone to transformations in an aqueous environment, undergoing processes such as hydrolysis, ligand exchange, and redox reactions. In the case of vanadium­(V) compounds, redox interconversions may occur in a cellular environment; in fact, V^V^–L complexes may be reduced to V^IV^ by various reducing agents in the cytosol where the resulting V^IV^O^2+^ ion can be coordinated not only by the original ligand L but also by the reductants themselves or by other endogenous bioligands.^1,45^ These processes can lead to the formation of multiple adducts or mixtures thereof, which may collectively contribute to the observed biological effects. Identifying the active species in the organism is one of the factors that has slowed the development of vanadium drugs by pharmaceutical companies and that could suggest more rational strategies for the design of vanadium-based therapeutics.^46^

Among the possible reducing agents of the cytosol, we investigated ascorbate (Asc) and glutathione (GSH). The cytosolic concentration of Asc is in the range ∼0.1–10 mM, even if often a more restricted range, ∼0.5–5 mM, is reported,^47,48^ while that of GSH is between 1 and 10 mM and can reach up to 15 mM.^49,50^ For many years, GSH was considered the main species responsible for the reduction of vanadium­(V) complexes, but subsequent studies have questioned this view, and the literature currently reports conflicting results. While some authors have shown that V^V^ can be reduced by GSH,^51,52^ others have found that this process is negligible.^53^ For example, Asc is more effective than GSH in promoting the reduction of pharmacologically active V^V^ complexes formed by maltolate and picolinate.^54–56^ Some reports also suggest that V^IV^ species may be more biologically active than V^V^ compounds, and that the reduction of vanadate­(V) by ascorbate or thiol-containing ligands can potentiate the biological activity of VCs.^57,58^

In this work, EPR experiments were performed on solutions containing Cs_2_[V^V^_2_O_4_(lact)_2_]·2H_2_O in PBS at pH 7.4 to determine if the reduction occurs and which V^IV^O complexes can form. An excess of reducing agent was used with a reductant-to-VC molar ratio set to 10:1. EPR measurements revealed that, over time, V^V^ is progressively converted into EPR-active V^IV^ species. As illustrated in Figure 7, the intensity of the spectral signal, proportional to the V^IV^ concentration, increases rapidly at first, reaching a maximum after 5–8 h.

**Figure 7:**
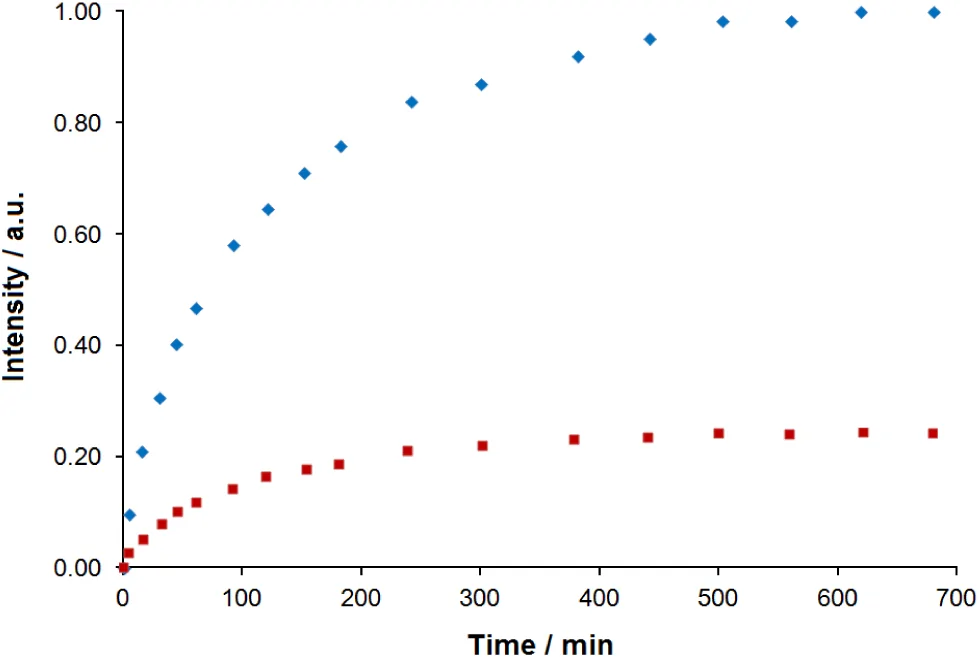
Variation of the EPR spectral intensity (in arbitrary units, a.u.) as a function of time (in min) in the systems containing [V^V^_2_O_4_(lact)_2_]^2–^ and Asc (blue rhombi) or GSH (red squares). The molar ratio [V^V^_2_O_4_(lact)_2_]^2–^/Asc and [V^V^_2_O_4_(lact)_2_]^2–^/GSH is 1:10.

Data from the reduction process are strongly dependent on the reducing agent and suggest that Asc is slightly more effective than GSH in the generation of V^IV^, in agreement with previously published papers.^12,56^ With an increase in the strength of the reducing agent, the amount of the reduced species also increases, delaying the time at which the maximum concentration of V^IV^ is reached.

EPR spectra recorded on the system [V^V^_2_O_4_(lact)_2_]^2–^/Asc 1:10 show that, with the elapsing time, the signals of two V^IV^O species appear. One of them, in a lower amount, indicated as **I** in Figure 8, is the monochelated complex [V^IV^O­(lact)­(H_2_O)_2_] with (COO^–^, O^–^) binding mode of lactate and two aqua ligands on the two remaining equatorial positions; its spin Hamiltonian parameters are *g*_*z*_ = 1.942 and *A*_*z*_ = 168.4 × 10^–4^ cm^–1^, in line with what was reported previously.^59^ The second species, formed in solution in a higher amount, is [V^IV^O­(lact)_2_]^2–^ (**II** in Figure 8). It has a 2 × (COO^–^, O^–^) coordination mode, and *g*_*z*_ = 1.951 and *A*_*z*_ = 156.4 × 10^–4^ cm^–1^, in good agreement with the literature data.^59^ These results suggest that Asc, being a weak ligand, does not take part in the metal coordination at physiological pH; moreover, the formation of mixed-ligand species is not observed. The data agree well with the prediction based on the stability constants for the V^IV^O–lact complexes for a V^IV^ concentration of 4 mM, a metal-to-ligand molar ratio of 1:1 and pH 7.4, which indicate a higher percent concentration of [V^IV^O­(lact)_2_]^2–^ than [V^IV^O­(lact)­(H_2_O)_2_] under these experimental conditions.^60^

**Figure 8:**
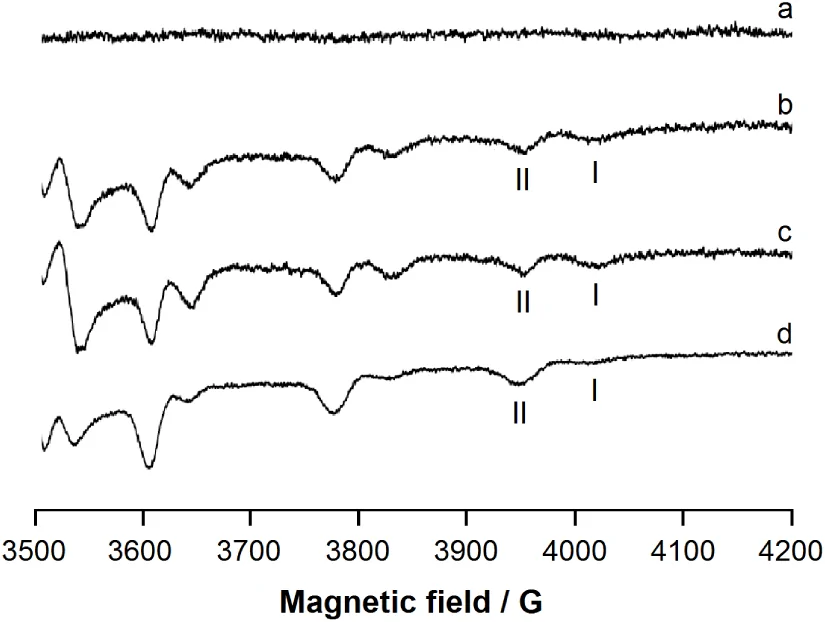
High-field region of the anisotropic EPR spectra recorded on the system [V^V^_2_O_4_(lact)_2_]^2–^/Asc 1:10 in PBS at pH 7.4 with a V concentration of 4 mM: (a) *t* = 0 min; (b) *t* = 40 min; (c) *t* = 120 min; (d) *t* = 300 min. **I**, [V^IV^O­(lact)­(H_2_O)_2_] with equatorial coordination (COO^–^, O^–^); 2 H_2_O; **II**, [V^IV^O­(lact)_2_]^2–^ with equatorial coordination 2 × (COO^–^, O^–^).

The EPR spectra of the system containing [V^V^_2_O_4_(lact)_2_]^2–^ and GSH with a molar ratio of 1:10 show a different behavior (Figure 9). The coexistence of four species is detected, indicated as **I**–**IV**. **I** and **II** are the 1:1 and 1:2 binary complexes formed by lactate, [V^IV^O­(lact)­(H_2_O)_2_] and [V^IV^O­(lact)_2_]^2–^, discussed above for the system with ascorbate. The other two species are formed by GSH (**III** and **IV** in Figure 9). The first one is [V^IV^O­(GSH)_2_]^2–^ (**III**) with equatorial coordination 2 × (NH_2_, COO^–^), typical of amino acids without coordinating side chains; its EPR parameters are *g*_*z*_ = 1.951 and *A*_*z*_ = 162.8 × 10^–4^ cm^–1^, in agreement with the literature outcomes.^61,62^ The second complex formed by GSH is characterized, compared to [V^IV^O­(GSH)_2_]^2–^, by two additional deprotonations and its stoichiometry is [V^IV^O­(GSH)­(GSHH_–2_)]^4–^ (**IV**). Its spin Hamiltonian parameters are *g*_*z*_ = 1.961 and *A*_*z*_ = 154.4 × 10^–4^ cm^–1^. Its binding mode has not been definitively determined: on one hand, it has been described by Dessì et al. as a mixed coordination with (NH_2_, COO^–^) for the first GSH ligand and (N_amide_^–^, S^–^) for the second one;^61^ however, other authors have proposed different coordination modes but all with the deprotonation of the amide group.^62–64^ For example, Costa Pessoa et al. suggested a stoichiometry [V^IV^O­(GSHH_–2_)­(H_2_O)]^2–^ and a binding mode (NH_2_, N_amide_^–^, S^–^); H_2_O.^62^ Overall, for the system [V^V^_2_O_4_(lact)_2_]^2–^/GSH, the experimental findings indicate that (i) V^IV^O^2+^ ion distributes among more than one ligand, (ii) GSH is a stronger ligand than Asc; and (iii) no mixed-ligand complexes are formed. If the stability constants of the V^IV^O–lact and V^IV^O–GSH complexes^60,62^ are considered, a comparable amount of [V^IV^O­(lact)_2_]^2–^, [V^IV^O­(GSH)_2_]^2–^, and [V^IV^O­(GSHH_–2_)­(H_2_O)]^2–^ is predicted, in good agreement with the spectra depicted in Figure 9.

**Figure 9:**
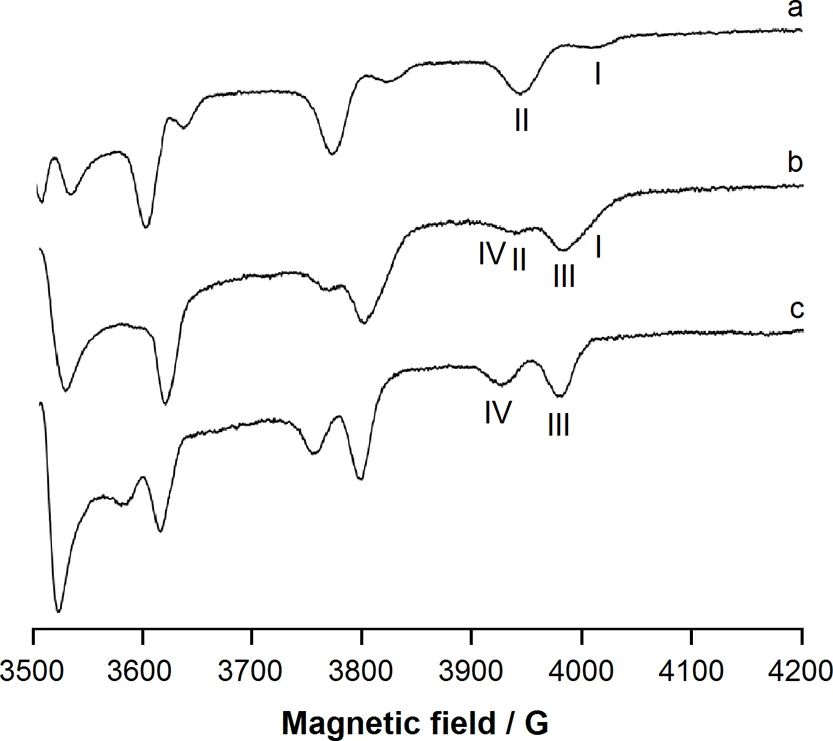
High-field region of the anisotropic EPR spectra recorded on the systems: (a) V^IV^O^2+^/lact 1:1, pH 7.4, and V concentration 4 mM; (b) [V^V^_2_O_4_(lact)_2_]^2–^/GSH 1:10 in PBS, pH 7.4, and V concentration of 4 mM; (c) V^IV^O^2+^/GSH 1:25, pH 7.4, and V concentration 4 mM. **I**, [V^IV^O­(lact)­(H_2_O)_2_] with equatorial coordination (COO^–^, O^–^); 2 H_2_O; **II**, [V^IV^O­(lact)_2_]^2–^ with equatorial coordination 2 × (COO^–^, O^–^); **III**, [V^IV^O­(GSH)_2_]^2–^ with equatorial coordination 2 × (NH_2_, COO^–^); **IV**, [V^IV^O­(GSHH_–2_)­(H_2_O)]^2–^ with equatorial coordination (NH_2_, N_amide_^–^, S^–^); H_2_O.

### ROS Formation

VCs exhibit redox properties that can modulate cell signaling and enhance the glucose uptake. They can also induce oxidative stress through the generation of reactive oxygen species (ROS), leading to damage to biomolecules and, ultimately, cell death.^65^ In this context, some studies suggest that the intrinsic lability and redox reactivity of VCs in biological environments could be advantageous for therapeutic applications.^45,66,67^ The generation of ROS promotes many processes among which are the cleavage of DNA and the inhibition of phosphotyrosine phosphatases (PTPases).^1,2,68^ As highlighted in the literature, modulation of ROS levels by VCs may also have therapeutic potential in diseases associated with oxidative imbalance.^3^

Vanadium-induced ROS formation is primarily due to the V^IV^/V^V^ redox cycle, which first promotes the generation of superoxide anion (O_2_^–•^) and then of hydrogen peroxide (H_2_O_2_), with a subsequent conversion to highly reactive hydroxyl radicals (^•^OH) via Fenton-like processes. The key species in these processes is V^IV^ which initially leads to the formation of O_2_^–•^ through the reaction V^IV^ + O_2_ → V^V^ + O_2_^–•^; after, O_2_^–•^ is transformed into H_2_O_2_ by 2 O_2_^–•^ + 2 H^+^ → H_2_O_2_ + O_2_. The formation of most damaging ROS, ^•^OH, is the result of the reaction of V^IV^ with H_2_O_2_ in a Fenton-like process: V^IV^O^2+^ + H_2_O_2_ → V^V^O^3+^ + ^•^OH + OH^–^.^3,69^ The Fenton reaction is given obviously by Fe^2+^ whose intracellular concentration is tightly regulated to prevent this harmful chemical transformation.^70^ Other metal ions that can promote similar Fenton-like processes are Cu^+^, Co^2+^, and Ti^3+^. These pathways involve redox-active metal ions, and ^•^OH formation is enhanced by biological reductants such as glutathione, nicotinamide adenine dinucleotide (NADH), ascorbate, and cysteine, and is associated with the disruption of the mitochondrial electron transport chain.

In this study, the formation of the radical ^•^OH was monitored by EPR spectroscopy, trapping it with DMPO to form a stable radical adduct DMPO–OH (Figure S1). The amount of the adduct DMPO–OH can be measured by the intensity of the EPR signal, which is due to the coupling of the unpaired electron with the nuclei of ^14^N (*I* = 1) and ^1^H (*I* = 1/2) with a comparable *A*_iso_ values (*A*_iso_(^14^N) = 14.5 G and *A*_iso_(^1^H) = 13.2 G), resulting in a four-line spectrum with an intensity ratio between the resonances of 1:2:2:1. The spectrum of DMPO–OH is given in Figure S8.

Initially, the systems containing [V^V^_2_O_4_(lact)_2_]^2–^ and those with V^V^O_2_^+^ and lactate were examined. Afterward, the systems with the dinuclear V^V^–lactate complex plus GSH or Asc were also studied. Finally, the production of radicals was compared with that induced by Fe^2+^, by the free V^IV^O^2+^ ion, and by a “benchmark” complex, i.e., [V^IV^O­(acac)_2_], where acac is the acetylacetonate ligand. The experiments with free metal ions were performed at acidic pH (∼3) to prevent hydroxide precipitation and avoid the complexation of the ions by the OH^–^ ligand at physiological pH.

The EPR spectra in Figure 10 show that, under the same experimental conditions, the formation of ROS with lactate, [V^V^_2_O_4_(lact)_2_]^2–^ and V^V^O_2_^+^ is close to zero. Lactate is not intrinsically a pro-oxidant and does not promote Fenton-type chemistry. The same conclusion is valid for V^V^O_2_^+^ and [V^V^_2_O_4_(lact)_2_]^2–^ since a Fenton-like reaction does not involve the highest oxidation state of a metal ion. The production of ^•^OH by V^V^–lact species increases significantly when GSH or Asc are present in the solution (see below).

**Figure 10:**
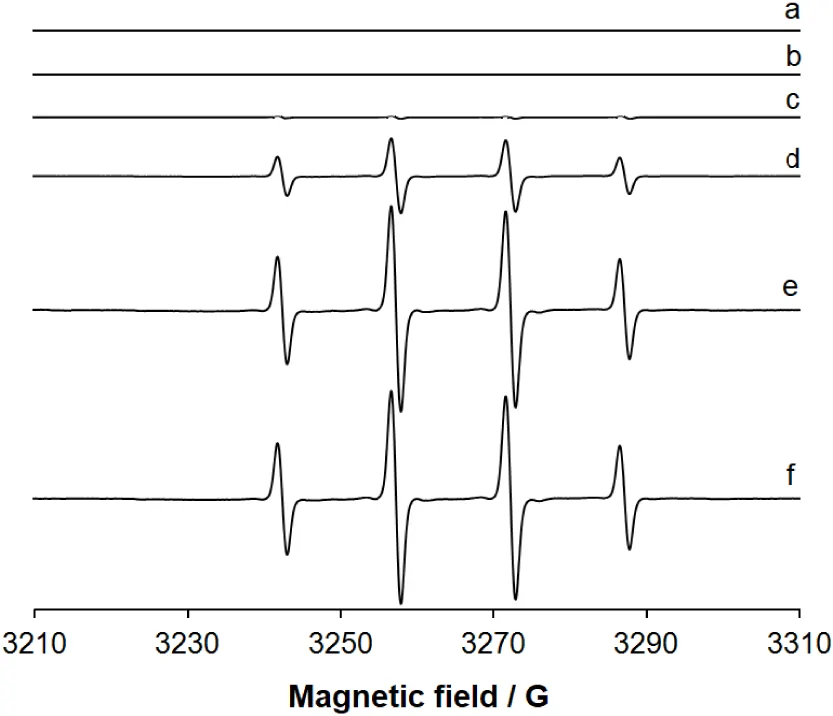
Experimental EPR spectrum of the adduct DMPO–OH revealed in the systems containing: (a) lactate; (b) free V^V^O_2_^+^; (c) complex [V^V^_2_O_4_(lact)_2_]^2–^; (d) [V^V^_2_O_4_(lact)_2_]^2–^ in the presence of Asc; (e) free V^IV^O^2+^; (f) free Fe^2+^. The concentration of the metal ion was always 2.5 × 10^–5^ M and pH was 3 for the spectra in traces b, e, and f and 7.4 in all the other ones. The ratio Asc/V^V^ in trace d was 5:1.

The amount of DMPO–OH adduct formed is reported in Figure 11, where the comparison between the systems [V^V^_2_O_4_(lact)_2_]^2–^/GSH, [V^V^_2_O_4_(lact)_2_]^2–^/Asc, Fe^2+^, V^IV^O^2+^, and [V^IV^O­(acac)_2_] is presented.

**Figure 11:**
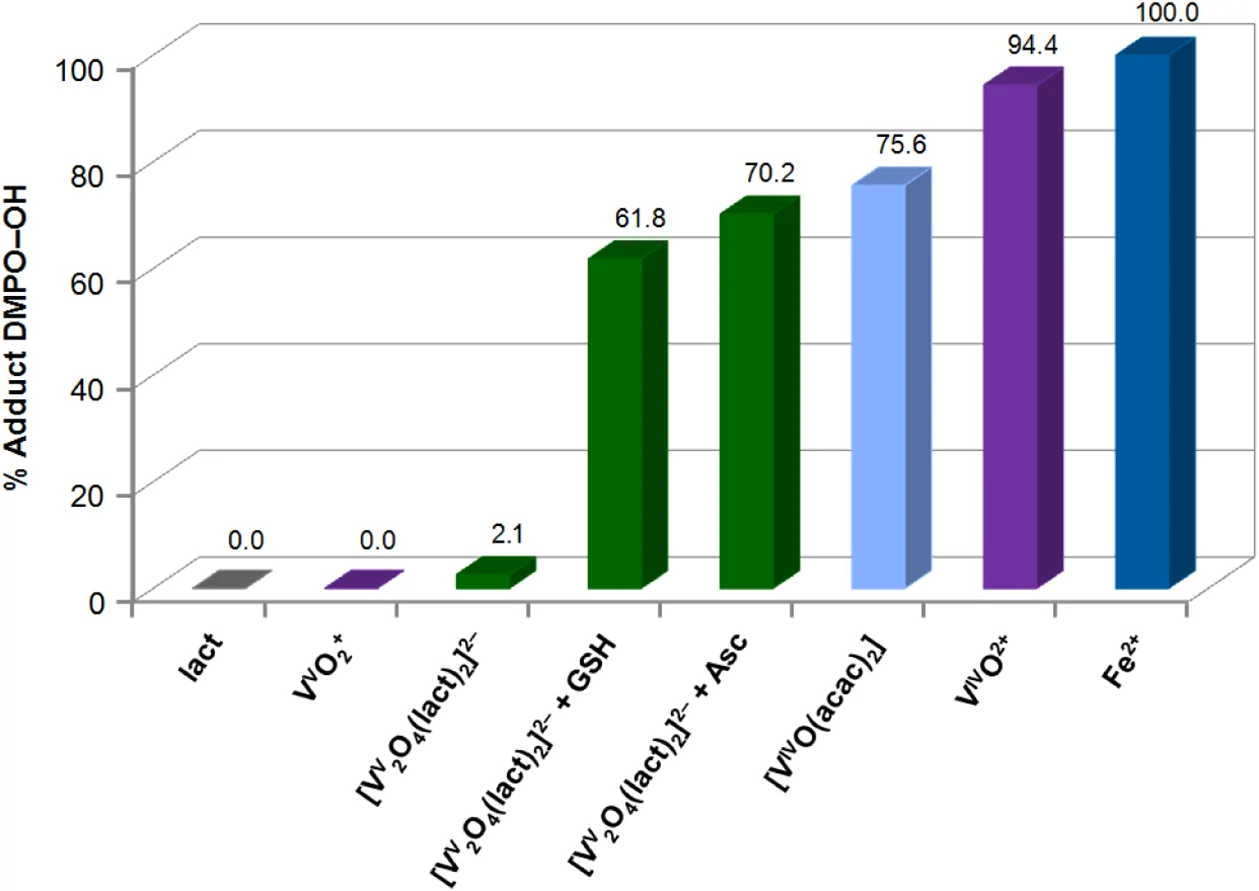
Percentage of DMPO–OH adduct formed in the systems containing from the left: lact, free V^V^O_2_^+^, [V^V^_2_O_4_(lact)_2_]^2–^, [V^V^_2_O_4_(lact)_2_]^2–^ plus GSH, [V^V^_2_O_4_(lact)_2_]^2–^ plus Asc, [V^IV^O­(acac)_2_], free V^IV^O^2+^, and free Fe^2+^. Experimental conditions: the concentration of lact and all the metal species was 2.5 × 10^–5^ M, the H_2_O_2_ and DMPO concentrations were 1.0 × 10^–4^ M and 2.925 × 10^–3^ M, respectively; pH was *ca*. 3 for the solutions containing V^IV^O^2+^, V^V^O_2_^+^ or Fe^2+^ ion, and 7.4 in all the other cases; in the two systems with a reductant, the molar ratio between Asc and GSH and [V^V^_2_O_4_(lact)_2_]^2–^ was 5:1.

If the systems with V^IV^O^2+^ and Fe^2+^ are considered, when the metal concentration is 2.5 × 10^–5^ M, the amount of DMPO–OH adduct, monitored by EPR signals, is lower in the first case than in the second one (signals with V^IV^O^2+^ reach ∼94% compared with those due to Fe^2+^). Overall, the data demonstrate that the aqua ion [V^IV^O­(H_2_O)_5_]^2+^ is at least as effective as [Fe­(H_2_O)_6_]^2+^ in driving Fenton-type processes, and this result appears to be in agreement with previously published studies which unambiguously demonstrated the ability of V^IV^ to promote Fenton-like reactions.^71–74^ Instead, the DMPO–OH signal intensity produced by [V^IV^O­(acac)_2_] is ∼75% of that observed for Fe^2+^ (Figure 11), the lower efficiency of [V^IV^O­(acac)_2_] in promoting Fenton-like reactions being attributed to the complexation effect and stabilization of the V^IV^ state, in line with literature data.^74^

As mentioned above, while the complex [V^V^_2_O_4_(lact)_2_]^2–^ does not produce ^•^OH radicals in an appreciable amount, the formation of ^•^OH significantly increases if the experiments are repeated in the presence of a reducing agent such as GSH or Asc. The data indicate that the percentage amount of the adduct DMPO–OH is between *ca.* 60 and 70% compared to Fe^2+^ taken as a reference (Figure 11). This means that the reduction of V^V^O_2_^+^ to V^IV^O^2+^ is the key step in the process and that—as pointed out in the literature—the formation of the ^•^OH radicals does not derive directly from V^V^ but can be related to the reduction to V^IV^. Moreover, the results suggest that Asc is slightly more effective as a reducing agent than GSH in the production of ^•^OH, confirming the established results^12,56^ and the data presented above for the reduction of [V^V^_2_O_4_(lact)_2_]^2–^ in an aqueous solution.

Therefore, upon cellular uptake, the partial reduction of [V^V^_2_O_4_(lact)_2_]^2–^ to mononuclear V^IV^O–lact species (discussed in the previous section on the reduction of [V^V^_2_O_4_(lact)_2_]^2–^), promoted by endogenous reductants such as GSH or Asc, is the basis of production of ROS, and in particular of hydroxyl radicals ^•^OH through Fenton-like reactions. The lower amount of ^•^OH produced by [V^V^_2_O_4_(lact)_2_]^2–^ in a reducing environment with respect to that formed by free V^IV^O^2+^ could be related to the stabilization of the V^IV^ center upon complexation by a strong lactate ligand. However, the presence of lactate may further influence these processes by modulating reduction kinetics and affecting intracellular localization, thereby impacting ROS yield and cytotoxic activity. Moreover, it must be observed that, if on one hand lactate lowers the amount of generated ^•^OH, on the other it might increase the lipophilicity of the V^IV^O complexes, facilitating the cellular uptake. Overall, these data account for the cytotoxic activity found for [V^V^_2_O_4_(lact)_2_]^2–^, which is higher than that of cisplatin for some types of cell lines, such as HaCaT, BALB/c-3T3, and PC-3 (Table 4).

### In-Cell ROS Generation Studies

To further support EPR analyses, intracellular ROS levels were evaluated by the DCFDA assay^75^ in both tumor cell lines after 1, 6, and 24 h of treatment with both dinuclear [V^V^_2_O_4_(mal)_2_]^2–^ and [V^V^_2_O_4_(lact)_2_]^2–^ complexes at their respective IC_50_ concentrations. In HeLa cells (Figure 12A), both complexes induced a rapid increase in intracellular ROS levels at 1 h of treatment (about 300%) compared with untreated control cells. High intracellular ROS levels were maintained throughout the experiment, showing a slight time-dependent increase observed up to 24 h. In PC-3 cells (Figure 12B), the treatment with the two complexes exhibited a similar trend, with ROS progressively increasing, reaching a maximum at 6 h (about 300% for [V^V^_2_O_4_(mal)_2_]^2–^ complex and about 250% for [V^V^_2_O_4_(lact)_2_]^2–^ complex), followed by a marked decline at 24 h. The difference observed between HeLa and PC-3 cells likely reflects a higher sensitivity of PC-3 cells to VCs. Overall, these findings suggest that ROS generation is an early event that may contribute to the activation of downstream cell death pathways.

**Figure 12:**
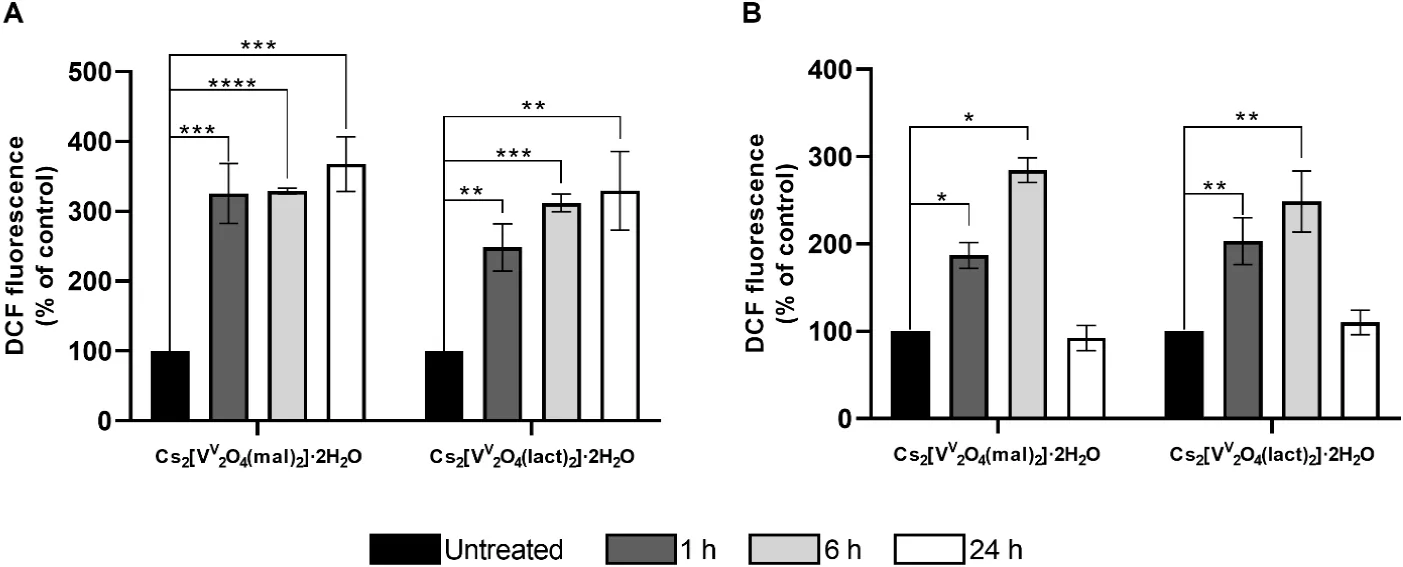
Determination of intracellular ROS levels by DCFDA assay. A) HeLa and B) PC-3 cells were incubated for 1 h (dark gray bars), 6 h (light gray bars), or 24 h (white bars) with IC_50_ concentrations of Cs_2_[V^V^_2_O_4_(mal)_2_]·2H_2_O or Cs_2_[V^V^_2_O_4_(lact)_2_]·2H_2_O. Values are expressed as percentages compared with untreated cells (black bars). * indicates *p* < 0.05, ** indicates *p* < 0.01, *** indicates *p* < 0.001, and **** indicates *p* < 0.0001. Lines above bars indicate the samples compared for statistical analysis.

## Conclusions

Vanadium compounds show promising anticancer activities, and for this reason, an increasing amount of research is focused on their synthesis, characterization, and in-solution behavior. It is well established that, in aqueous media, VCs undergo a variety of processes, including ligand exchange, hydrolysis, and redox reactions, which are governed by the properties of the compounds, metal concentration, pH, and interaction with bioligands such as reducing agents and proteins.^1^ As a result, when pharmacologically active VCs are introduced into biological fluids, the predominant species in solution may differ from the initially administered compound and, therefore, a detailed understanding of their behavior and transformation in biological environments and interaction with proteins is crucial in order to identify the active species in the organism and elucidate the mechanism of action.

This study provides a comprehensive description of the chemical and biological behavior of the dioxidovanadium­(V) complex formed by lactate, Cs_2_[V^V^_2_O_4_(lact)_2_]·2H_2_O, with promising cytotoxic activity. The results demonstrate that this compound does not retain its molecular structure in solution and, in the presence of a protein such as lysozyme, undergoes extensive transformation, generating a complex distribution of mono-, di-, and polynuclear vanadium species whose relative abundance is strongly influenced by pH and experimental conditions. The comparison with the similar system with malate suggests that even closely related α-hydroxycarboxylate ligands can significantly modulate vanadium speciation and reactivity, with important consequences for biological behavior. Protein binding studies reveal that multiple vanadium-containing fragments interact with lysozyme through noncovalent interactions, and that the protein environment selectively stabilizes specific metal moieties and suppresses others, such as decavanadate. The overall protein structure remains unaffected in the presence of the VC. Notably, for the first time, the formation of adducts between a protein, cyclic [V^V^_3_O_9_]^3–^, and [V^V^_3_O_7_(lact)_2_]^3–^ anion has been observed, confirming the dynamic nature of vanadium speciation in biological systems.

A key outcome of this work is the elucidation of the redox behavior of the studied V^V^–lactate complex. EPR experiments clearly show that biologically relevant reductants such as ascorbate and GSH promote the reduction of V^V^ to V^IV^, leading to the formation of V^IV^O–lactate and V^IV^O–GSH species that could represent some of the active forms in a cellular environment. Importantly, the reduction to vanadium­(IV) is the critical step for the activation of Fenton-like processes which result in the formation of hydroxyl radicals ^•^OH, indicating that V^IV^ species are efficient promoters of oxidative stress. The lactate ligand plays a dual role: on one hand, it modulates the redox properties and limits excessive radical production by stabilizing the reduced V^IV^ form; on the other, it likely enhances the cellular uptake of Cs_2_[V^V^_2_O_4_(lact)_2_]·2H_2_O, thereby contributing to its biological activity. In this scenario, the observed cytotoxicity can be rationalized in terms of an intracellular reduction process followed by ROS generation and oxidative damage. The increase in ROS levels in HeLa and PC-3 cells in the presence of Cs_2_[V^V^_2_O_4_(lact)_2_]·2H_2_O and Cs_2_[V^V^_2_O_4_(mal)_2_]·2H_2_O has been indeed verified. In any case, we cannot establish which is the active species responsible for this behavior, if there is more than one species involved in the processes, and we cannot exclude that other reactions could play a role in the cell death.

Overall, this work highlights once again that the biological activity of VCs arises from a delicate balance between speciation, redox transformations, and interactions with biomolecules, all of these factors providing mechanistic insights into their action mode and underscoring the importance of controlling the ligand environment and redox chemistry for a rational design of V-based therapeutics.

## Supplementary Material



## Notes

List of abbreviations and acronyms used in this work; data collection and refinement statistics for structures of the adduct formed upon reaction of Cs_2_[V^V^_2_O_4_(lact)_2_]·2H_2_O with lysozyme (structures **A–D**) (Table S1); chemical shifts in ^51^V NMR spectra measured in triplicate at RT after 1 h and 7 d of incubation of Cs_2_[V^V^_2_O_4_(lact)_2_]·2H_2_O in the absence and presence of lysozyme (Tables S2–S4); adduct formation between the hydroxyl radical (^•^OH) and DMPO (Figure S1); ^51^V NMR spectra recorded at RT after 1 h and 7 d of incubation of Cs_2_[V^V^_2_O_4_(lact)_2_]·2H_2_O in the absence and presence of lysozyme (Figures S2–S4); ESI mass spectra of Cs_2_[V^V^_2_O_4_(lact)_2_]·2H_2_O (Figure S5); CD spectra of HEWL in the absence and presence of Cs_2_[V^V^_2_O_4_(lact)_2_]·2H_2_O after 1 h, 48 h and 7 d of incubation (Figure S6); effect of vanadium complexes on cell viability (Figure S7); experimental EPR spectrum of the adduct DMPO–OH (Figure S8) (PDF)
