# Supplementary material for: Speciation, Protein Binding, Biotransformation, and Cytotoxicity of a VV–Lactate Complex
Source: Inorg Chem. 2026 Aug 15;65(34):19783–98. doi: 10.1021/acs.inorgchem.6c02377 (PMC13536881; doi:10.1021/acs.inorgchem.6c02377)
Supplement: Supplementary file 1 [file ic6c02377_si_001.pdf]

Supporting information

Speciation, protein binding, biotransformation and cytotoxicity of a V^V–lactate complex

Maddalena Paolillo,[a] Virginia Cuomo,[a] Giarita Ferraro,[a] Paola Imbimbo,[a] Nadiia I. Gumerova,[b] Federico Pisanu,[c] Eugenio Garribba,*[c] Annette Rompel,*[b] and Antonello Merlino*[a]

a Department of Chemical Sciences, University of Naples Federico II, Complesso Universitario di Monte Sant'Angelo, Via Cintia, I-80126, Napoli, Italy

E-mail: antonello.merlino@unina.it

b Universität Wien, Fakultät für Chemie, Institut für Biophysikalische Chemie, 1090 Wien, Austria

E-mail: annette.rompel@univie.ac.at; www.bpc.univie.ac.at

c Dipartimento di Medicina, Chirurgia e Farmacia, Università di Sassari, Viale San Pietro, I-07100 Sassari, Italy

E-mail: garribba@uniss.it

List of Abbreviations

Abbreviation	Definition
a.u.	arbitrary units
acac	acetylacetonate
ACN	acetonitrile
Asc	ascorbate
ATCC	American Type Culture Collection
BALB/c-3T3	immortalized murine fibroblast cell line
CCDC	Cambridge Crystallographic Data Centre
D_2O	deuterium oxide (heavy water)
DMEM	Dulbecco's Modified Eagle Medium
DMPO	5,5-dimethyl-1-pyrroline *N*-oxide
EG	ethylene glycol
EPR	electron paramagnetic resonance
ESI-MS	electrospray ionisation-mass spectrometry
ESRF	European Synchrotron Radiation Facility
EtOH	ethanol
FA	formic acid
GSH	glutathione (reduced)
HaCaT	immortalized human keratinocyte cell line
HeLa	human cervical carcinoma cell line
HEPES	4-(2-hydroxyethyl)-1-piperazineethanesulfonic acid
HEWL	hen egg white lysozyme
IC50	half maximal inhibitory concentration
lact	lactate
mal	malate
MeOH	methanol
MTT	3-(4,5-dimethylthiazol-2-yl)-2,5-diphenyltetrazolium bromide
NADH	nicotinamide adenine dinucleotide (reduced)
NMR	nuclear magnetic resonance
NZ	ε-amino group nitrogen atom of lysine (PDB nomenclature)
PBS	phosphate-buffered saline
PC-3	human prostate cancer cell line
PDB	Protein Data Bank
ppm	parts per million
PTPases	phosphotyrosine phosphatases
ROS	reactive oxygen species
RPMI	Roswell Park Memorial Institute medium (RPMI-1640)
RT	room temperature
SD	standard deviation
SI	Supporting Information
^{51}V NMR	vanadium-51 nuclear magnetic resonance
V_{10}	decavanadate, $[V_{10}O_{28}]^{6-}$
VCs	vanadium compounds

Table S1. Data collection and refinement statistics of structures **A-D**.

**Data collection**				
	Structure **A**	Structure **B**	Structure **C**	Structure **D**
**Crystallization condition**	1.1 M sodium chloride, 0.1 M sodium acetate, pH 4.0	20% ethylene glycol, 0.6 M sodium nitrate, 0.1 M sodium acetate, pH 4.0	0.8 M succinic acid, pH 7.0	2.0 M sodium formate, 0.1 M HEPES, pH 7.5
**PDB code**	30JS	30JU	30JX	30JY
**Soaking time**	7 d	7 d	7 d	7 d
**Space group**	$P4_32_12$	$P4_32_12$	$P4_32_12$	$P4_32_12$
**a (Å)**	77.85	77.10	76.34	76.41
**b (Å)**	77.85	77.10	76.34	76.41
**c (Å)**	37.09	37.41	37.01	36.98
**Resolution range (Å)**	55.05-1.20 (1.22-1.20)	38.55-1.34 (1.37-1.34)	54.00-1.49 (1.51-1.49)	54.03-1.23 (1.25-1.23)
**Observations**	841373 (36231)	303896 (7644)	194724 (9282)	273116 (3394)
**Unique reflections**	36597 (1770)	25659 (1243)	18528 (893)	30853 (1100)
**Completeness (%)**	100.0 (100.0)	100.0 (100.0)	99.9 (100.0)	95.3 (69.6)
**Redundancy**	23.0 (20.5)	11.8 (6.1)	10.5 (10.4)	8.9 (3.1)
**Rmerge (%)**	0.073 (1.800)	0.116 (2.114)	0.148 (4.604)	0.084 (1.096)
**Average I/σ(I)**	19.7 (2.1)	7.8 (0.5)	7.7 (0.6)	12.6 (1.3)
**$CC_{1/2}$**	0.999 (0.856)	0.998 (0.378)	0.996 (0.398)	0.998 (0.331)
**Anom. completeness (%)**	100.0 (100.0)	100.0 (99.2)	100.0 (100.0)	91.0 (44.9)
**Anom. Multiplicity**	12.2 (10.6)	6.3 (3.2)	5.7 (5.5)	4.9 (2.0)
**Refinement**				
**Resolution (Å)**	55.05-1.20	55.31-1.36	54.01-1.52	54.03-1.23
**N° reflections**	34372	22388	15203	28383
**N° reflections in working set**	2504	418	277	1030
**R-factor/Rfree**	0.174/0.201	0.174/0.217	0.175/0.227	0.180/0.218
**N° non-H atoms in the refinement**	1256	1272	1177	1231
**B-factor overall (Å²)**	21.04	20.79	22.69	17.48
**Estimated occupancy of $[V^VO_2]^+$**	0.70	0.50	-	-
**Estimated occupancy of $[V^V_2O_4(lact)_2]^{2-}$**	0.80	0.80	0.80	-
**Estimated occupancy of $[V^V_3O_9]^{3-}$**	-	0.50	-	-
**Estimated occupancy of $[V^{IV}O]^{2+}$**	-	-	0.40	0.40
**Estimated occupancy of $[V^{IV}O]^{2+}$**	-	-	0.40	0.40
**Estimated occupancy of $[V^V_3O_7(lact)_2]^{3-}$**	-	-	0.60	0.60
**B-factor of $[V^VO_2]^+$ (Å²)**	40.69	37.66	-	-
**B-factor of $[V^V_2O_4(lact)_2]^{2-}$ (Å²)**	23.17±3.40	28.74±4.04	36.48±3.20	-
**B-factor of $[V^V_3O_9]^{3-}$ (Å²)**	-	38.55±5.14	-	-
**B-factor of $[V^{IV}O]^{2+}$ (Å²)**	-	-	22.78	24.99
**B-factor of $[V^{IV}O]^{2+}$ (Å²)**	-	-	32.33	29.15
**B-factor of $[V^V_3O_7(lact)_2]^{3-}$ (Å²)**	-	-	34.47±2.43	28.81±4.13
**Ramachandran values (%)**				
**Most favoured/Additional allowed**	93.75/6.25	94.64/5.36	95.97/4.03	95.16/4.84
**Outliers**	0	0	1	0
**RMSD from ideality**				
**RMSD bonds (Å)**	0.014	0.012	0.010	0.012
**RMSD angles (°)**	1.908	1.920	4.157	4.171

Table S2. Chemical shifts in ^{51}V NMR spectra measured in triplicate at RT after 1 h and 7 d of incubation of $Cs_2[V^V{}_2O_4(lact)_2]\cdot 2H_2O$ (5 mM) dissolved in 0.1 M sodium acetate, pH 4.0, 20% ethylene glycol, 0.6 M sodium nitrate, 10% D_2O; and 0.1 M sodium acetate, pH 4.0, 20% ethylene glycol, 0.6 M sodium nitrate, 10% D_2O in presence of lysozyme (13 mg/mL). The relative content of species was calculated based on the integration of ^{51}V signals. Signals were assigned based on literature data. [a]

**5 mM $Cs_2[V^V{}_2O_4(lact)_2]\cdot 2H_2O$ in 0.1 M NaOAc, pH 4.0, 20% ethylene glycol, 0.6 M $NaNO_3$, 10% D_2O (after 1 h at RT)**											
**^{51}V chemical shift (ppm)**			**Species**	**References**	**Integration (%)**			**Sum**			**Average ± SD (%)**
**Sample 1**	**Sample 2**	**Sample 3**			**Sample 1**	**Sample 2**	**Sample 3**	**Sample 1**	**Sample 2**	**Sample 3**	
–425	–425	–425	V_{10}	[1,2]	0.1	0.1	0.1	14.9	14.8	20.5	16.7 ± 3.3
–502	–502	–502	V_{10} (or VL^-)	[1–6]	1.4	1.9	3.9				
–520	–520	–520	V_{10} (or VL^{2-})	[1–6]	13.4	12.8	16.5				
–523	–523	–523	$V_2EG_2{}^{2-}$	[7]	3.2	2.8	3.3	3.2	2.8	3.3	3.1 ± 0.3
–532	–532	–531	$V_2L_2{}^{2-}$	[1–6]	64.6	66.6	64.9	64.6	66.6	64.9	65.4 ± 1.1
–538	–538	–538	$V_3L_2{}^{3-}$ (or V_2L^{2-})	[1–6]	11.5	10.8	7.4	17.3	15.8	11.3	14.8 ± 3.1
–546	–546	–545	$V_3L_2{}^{3-}$ (or V_2L^{2-})	[1–6]	5.8	5.0	3.9				
**5 mM $Cs_2[V^V{}_2O_4(lact)_2]\cdot 2H_2O$ + 13 mg/mL lysozyme in 0.1 M NaOAc, pH 4.0, 20% ethylene glycol, 0.6 M $NaNO_3$, 10% D_2O (after 1 h at RT)**											
**^{51}V chemical shift (ppm)**			**Species**	**References**	**Integration (%)**			**Sum**			**Average ± SD (%)**
**Sample 1**	**Sample 2**	**Sample 3**			**Sample 1**	**Sample 2**	**Sample 3**	**Sample 1**	**Sample 2**	**Sample 3**	
–520	–520	–520	VL^{2-}	[1–6]	12.0	15.9	15.0	12.0	15.9	15.0	14.3 ± 2.0
–523	–523	–523	$V_2EG_2{}^{2-}$	[7]	4.9	5.0	3.6	4.9	5.0	3.6	4.5 ± 0.8
–531	–531	–531	$V_2L_2{}^{2-}$	[1–6]	69.8	70.6	73.2	69.8	70.6	73.2	71.2 ± 1.8
–538	–538	–538	$V_3L_2{}^{3-}$ (or V_2L^{2-})	[1–6]	9.3	5.8	5.6	13.3	8.5	8.2	10.0 ± 2.9
–545	–545	–545	$V_3L_2{}^{3-}$ (or V_2L^{2-})	[1–6]	4.0	2.7	2.6				
**5 mM $Cs_2[V^V{}_2O_4(lact)_2]\cdot 2H_2O$ in 0.1 M NaOAc, pH 4.0, 20% ethylene glycol, 0.6 M $NaNO_3$, 10% D_2O (after 7 d at RT)**											
**^{51}V chemical shift (ppm)**			**Species**	**References**	**Integration (%)**			**Sum**			**Average ± SD (%)**
**Sample 1**	**Sample 2**	**Sample 3**			**Sample 1**	**Sample 2**	**Sample 3**	**Sample 1**	**Sample 2**	**Sample 3**	
–421	–425	–425	V_{10}	[1,2]	1.2	0.3	0.3	10.5	10.1	9.8	10.1 ± 0.4
–502	–502	–502	V_{10}	[1,2]	7.4	7.1	7.3				
–519	–519	–519	V_{10}	[1,2]	1.9	2.7	2.2				
–509	–509	–509	VL^-	[1–6]	7.6	11.0	7.9	7.6	11.0	7.9	8.8 ± 1.9
–520	–520	–520	VL^{2-}	[1–6]	7.7	8.6	7.8	7.7	8.6	7.8	8.0 ± 0.5
–523	–523	–523	$V_2EG_2{}^{2-}$	[7]	5.2	6.0	4.8	5.2	6.0	4.8	5.3 ± 0.6
–531	–531	–531	$V_2L_2{}^{2-}$	[1–6]	57.1	54.3	57.7	57.1	54.3	57.7	56.4 ± 1.8
–538	–538	–538	$V_3L_2{}^{3-}$	[1–6]	8.1	7.1	8.1	11.9	10.0	12.0	11.3 ±

			(or V_2L^{2-})								1.1
–546	–545	–545	$V_3L_2^{3-}$ (or V_2L^{2-})	[1–6]	3.8	2.9	3.9				
**5 mM $Cs_2[V^V{}_2O_4(lact)_2]\cdot 2H_2O$ + 13 mg/mL lysozyme in 0.1 M NaOAc, pH 4.0, 20% ethylene glycol, 0.6 M $NaNO_3$, 10% D_2O (after 7 d at RT)**											
**^{51}V chemical shift (ppm)**			**Species**	**References**	**Integration (%)**			**Sum**			**Average ± SD (%)**
**Sample 1**	**Sample 2**	**Sample 3**			**Sample 1**	**Sample 2**	**Sample 3**	**Sample 1**	**Sample 2**	**Sample 3**	
–520	–520	–520	VL^{2-}	[1–6]	13.6	10.7	11.0	13.6	10.7	11.0	11.8 ± 1.6
–523	–523	–523	$V_2EG_2^{2-}$	[7]	3.5	4.9	3.1	3.5	4.9	3.1	3.8 ± 0.9
–531	–531	–531	$V_2L_2^{2-}$	[1–6]	69.8	70.2	69.8	69.8	70.2	69.8	69.9 ± 0.2
–538	–538	–538	$V_3L_2^{3-}$ (or V_2L^{2-})	[1–6]	9.3	10.4	11.4	13.1	14.2	16.1	14.5 ± 1.5
–545	–545	–546	$V_3L_2^{3-}$ (or V_2L^{2-})	[1–6]	3.8	3.8	4.7				

[a] L indicates $lact^{2-}$ ligand, VL^- indicates $[V^VO_2(lact)(H_2O)]^-$, VL^{2-} $[V^VO_2(lact)(OH)]^{2-}$, $V_2L_2^{2-}$ $[V^V{}_2O_4(lact)_2]^{2-}$, $V_2EG_2^{2-}$ $[V^V{}_2O_4(EG)_2]^{2-}$, V_2L^{2-} $[V^V{}_2O_5(lact)]^{2-}$ and $V_3L_2^{3-}$ $[V^V{}_3O_7(lact)_2]^{3-}$, d=days, RT=room temperature

Table S3. Chemical shifts in ^{51}V NMR spectra measured in triplicate at RT after 1 h and 7 d of incubation of $Cs_2[V^V_2O_4(lact)_2]\cdot 2H_2O$ (5 mM) dissolved in 0.8 M succinic acid, pH 7.0, 10% D_2O; and 0.8 M succinic acid, pH 7.0, 10% D_2O in presence of HEWL (13 mg/mL). The relative content of species was calculated based on the integration of ^{51}V signals. Signals were assigned based on literature data.[a]

**5 mM $Cs_2[V^V_2O_4(lact)_2]\cdot 2H_2O$ in 0.8 M succinic acid, pH 7.0, 10% D_2O (after 1 h at RT)**												
**^{51}V chemical shift (ppm)**			**Species**	**References**	**Integration (%)**			**Sum**			**Average ± SD (%)**
**Sample 1**	**Sample 2**	**Sample 3**			**Sample 1**	**Sample 2**	**Sample 3**	**Sample 1**	**Sample 2**	**Sample 3**	
–531	–532	–532	$V_2L_2^{2-}$	[1–6]	1.0	0.9	0.8	1.0	0.9	0.8	0.9 ± 0.1
–539	–540	–540	$V_3L_2^{3-}$ (or V_2L^{2-})	[1–6]	1.5	1.7	1.6	3.2	3.5	3.2	3.3 ± 0.2
–549	–549	–549	$V_3L_2^{3-}$ (or V_2L^{2-})	[1–6]	1.7	1.8	1.6				
–557	–555	–557	$[H_2V^VO_4]^-$	[6,8]	5.6	6.2	6.5	5.6	6.2	6.5	6.1 ± 0.5
–570	–570	–570	$[H_2V^V_2O_7]^{2-}$	[6,8]	3.0	2.0	2.9	3.0	2.0	2.9	2.6 ± 0.6
–574	–574	–574	$[V^V_4O_{12}]^{4-}$	[6,8]	71.5	69.8	70.6	71.5	69.8	70.6	70.6 ± 0.9
–583	–582	–582	$[V^V_5O_{15}]^{5-}$	[6,8]	15.7	17.6	16.0	15.7	17.6	16.0	16.4 ± 1.0

**5 mM $Cs_2[V^V_2O_4(lact)_2]\cdot 2H_2O$ + 13 mg/mL Lysozyme in 0.8 M succinic acid, pH 7.0, 10% D_2O (after 1 h at RT)**												
**^{51}V chemical shift (ppm)**			**Species**	**References**	**Integration (%)**			**Sum**			**Average ± SD (%)**
**Sample 1**	**Sample 2**	**Sample 3**			**Sample 1**	**Sample 2**	**Sample 3**	**Sample 1**	**Sample 2**	**Sample 3**	
–532	–531	–530	$V_2L_2^{2-}$	[1–6]	1.0	1.2	1.7	1.0	1.2	1.7	1.3 ± 0.4
–540	–539	–539	$V_3L_2^{3-}$ (or V_2L^{2-})	[1–6]	1.8	1.6	1.4	3.6	3.5	3.3	3.5 ± 0.2
–549	–548	–548	$V_3L_2^{3-}$ (or V_2L^{2-})	[1–6]	1.8	1.9	1.9				
–557	–556	–556	$[H_2V^VO_4]^-$	[6,8]	5.8	4.7	5.3	5.8	4.7	5.3	5.3 ± 0.6
–570	–570	–570	$[H_2V^V_2O_7]^{2-}$	[6,8]	3.4	4.3	5.9	3.4	4.3	5.9	4.5 ± 1.3
–574	–574	–574	$[V^V_4O_{12}]^{4-}$	[6,8]	69.4	71.0	68.7	69.4	71.0	68.7	69.7 ± 1.2
–582	–582	–582	$[V^V_5O_{15}]^{5-}$	[6,8]	16.8	15.3	15.1	16.8	15.3	15.1	15.7 ± 0.9

**5 mM $Cs_2[V^V_2O_4(lact)_2]\cdot 2H_2O$ in 0.8 M succinic acid, pH 7.0, 10% D_2O (after 7 d at RT)**												
**^{51}V chemical shift (ppm)**			**Species**	**References**	**Integration (%)**			**Sum**			**Average ± SD (%)**
**Sample 1**	**Sample 2**	**Sample 3**			**Sample 1**	**Sample 2**	**Sample 3**	**Sample 1**	**Sample 2**	**Sample 3**	
–531	–531	–532	$V_2L_2^{2-}$	[1–6]	1.0	1.0	0.8	1.0	1.0	0.8	0.9 ± 0.1
–539	–539	–540	$V_3L_2^{3-}$ (or V_2L^{2-})	[1–6]	1.8	2.0	1.8	3.7	3.9	3.6	3.7 ± 0.2
–548	–549	–549	$V_3L_2^{3-}$ (or V_2L^{2-})	[1–6]	1.9	1.9	1.8				
–557	–557	–557	$[H_2V^VO_4]^-$	[6,8]	5.3	4.8	5.1	5.3	4.8	5.1	5.1 ± 0.3
–570	–570	–570	$[H_2V^V_2O_7]^{2-}$	[6,8]	4.1	2.4	3.0	4.1	2.4	3.0	3.2 ± 0.9
–574	–574	–574	$[V^V_4O_{12}]^{4-}$	[6,8]	71.2	71.9	72.3	71.2	71.9	72.3	71.8 ± 0.6
–582	–582	–582	$[V^V_5O_{15}]^{5-}$	[6,8]	14.7	16.0	15.2	14.7	16.0	15.2	15.3 ± 0.7

**5 mM $Cs_2[V^V_2O_4(lact)_2]\cdot 2H_2O$ + 13 mg/mL Lysozyme in 0.8 M succinic acid, pH 7.0, 10% D_2O (after 7 d at RT)**												
**^{51}V chemical shift (ppm)**			**Species**	**Reference**	**Integration (%)**			**Sum**			**Averag**

Sampl e 1	Sampl e 2	Sampl e 3		s	Sampl e 1	Sampl e 2	Sampl e 3	Sampl e 1	Sampl e 2	Sampl e 3	e ± SD (%)
–532	–532	–531	$V_2L_2^{2-}$	1–6	1.3	1.3	1.2	1.3	1.3	1.2	1.3 ± 0.1
–540	–540	–539	$V_3L_2^{3-}$ (or V_2L^{2-})	1–6	3.2	2.5	2.6	6.5	5.3	5.5	5.8 ± 0.6
–549	–548	–549	$V_3L_2^{3-}$ (or V_2L^{2-})	1–6	3.3	2.8	2.9				
–557	–557	–557	$[H_2V^VO_4]^-$	6,8	5.7	4.7	5.7	5.7	4.7	5.7	5.4 ± 0.6
–570	–570	–570	$[H_2V^V{}_2O_7]^{2-}$	6,8	2.6	2.3	2.8	2.6	2.3	2.8	2.6 ± 0.3
–574	–574	–574	$[V^V{}_4O_{12}]^{4-}$	6,8	67.1	70.8	68.7	67.1	70.8	68.7	68.9 ± 1.9
–582	–5812	–582	$[V^V{}_5O_{15}]^{5-}$	6,8	16.8	15.6	16.1	16.8	15.6	16.1	16.2 ± 0.6

[a] L indicates $lact^{2-}$ ligand, VL^- indicates $[V^VO_2(lact)(H_2O)]^-$, VL^{2-} $[V^VO_2(lact)(OH)]^{2-}$, $V_2L_2^{2-}$ $[V^V{}_2O_4(lact)_2]^{2-}$, V_2L^{2-} $[V^V{}_2O_5(lact)]^{2-}$ and $V_3L_2^{3-}$ $[V^V{}_3O_7(lact)_2]^{3-}$.

Table S4. Chemical shifts in ^{51}V NMR spectra measured in triplicate at RT after 1 h and 7 d of incubation of $Cs_2[V^V_2O_4(lact)_2]\cdot 2H_2O$ (5 mM) dissolved in 0.1 M HEPES, pH 7.5, 2.0 M sodium formate and 10% D_2O; and 0.1 M HEPES, pH 7.5, 2.0 M sodium formate and 10% D_2O in presence of HEWL (13 mg/mL). The relative content of species was calculated based on the integration of ^{51}V signals. Signals were assigned based on literature data. [a]

**5 mM $Cs_2[V^V_2O_4(lact)_2]\cdot 2H_2O$ in 0.1 M HEPES, pH 7.5, 2.0 M sodium formate, 10% D_2O (after 1 h at RT)**											
**^{51}V chemical shift (ppm)**			**Species**	**References**	**Integration (%)**			**Sum**			**Average ± SD (%)**
**Sample 1**	**Sample 2**	**Sample 3**			**Sample 1**	**Sample 2**	**Sample 3**	**Sample 1**	**Sample 2**	**Sample 3**	
–539	–539	–539	$V_3L_2^{3-}$ (or V_2L^{2-})	1–6	0.6	0.6	1.0	1.4	2.0	2.5	2.0 ± 0.6
–549	–549	–549	$V_3L_2^{3-}$ (or V_2L^{2-})	1–6	0.8	1.4	1.5				
–555	–555	–556	$[H_2V^VO_4]^-$	6,8	4.9	5.5	3.7	4.9	5.5	3.7	4.7 ± 0.9
–570	–570	–570	$[H_2V^V_2O_7]^{2-}$	6,8	3.8	4.6	5.4	3.8	4.6	5.4	4.6 ± 0.8
–574	–574	–574	$[V^V_4O_{12}]^{4-}$	6,8	73.9	72.1	71.4	73.9	72.1	71.4	72.4 ± 1.3
–582	–582	–582	$[V^V_5O_{15}]^{5-}$	6,8	16.0	15.8	17.0	16.0	15.8	17.0	16.3 ± 0.6
**5 mM $Cs_2[V^V_2O_4(lact)_2]\cdot 2H_2O$ + 13 mg/mL HEWL in 0.1 M HEPES, pH 7.5, 2.0 M sodium formate, 10% D_2O (after 1 h at RT)**											
**^{51}V chemical shift (ppm)**			**Species**	**References**	**Integration (%)**			**Sum**			**Average ± SD (%)**
**Sample 1**	**Sample 2**	**Sample 3**			**Sample 1**	**Sample 2**	**Sample 3**	**Sample 1**	**Sample 2**	**Sample 3**	
–539	–539	–539	$V_3L_2^{3-}$ (or V_2L^{2-})	1–6	0.6	0.7	0.7	1.7	1.9	1.9	1.8 ± 0.1
–549	–549	–549	$V_3L_2^{3-}$ (or V_2L^{2-})	1–6	1.1	1.2	1.2				
–556	–556	–556	$[H_2V^VO_4]^-$	6,8	5.3	5.4	4.9	5.3	5.4	4.9	5.2 ± 0.3
–570	–570	–570	$[H_2V^V_2O_7]^{2-}$	6,8	4.0	5.4	4.0	4.0	5.4	4.0	4.5 ± 0.8
–574	–574	–574	$[V^V_4O_{12}]^{4-}$	6,8	74.0	71.2	73.7	74.0	71.2	73.7	73.0 ± 1.5
–582	–582	–582	$[V^V_5O_{15}]^{5-}$	6,8	15.0	16.1	15.5	15.0	16.1	15.5	15.5 ± 0.6
**5 mM $Cs_2[V^V_2O_4(lact)_2]\cdot 2H_2O$ in 0.1 M HEPES, pH 7.5, 2.0 M sodium formate, 10% D_2O (after 7 d at RT)**											
**^{51}V chemical shift (ppm)**			**Species**	**References**	**Integration (%)**			**Sum**			**Average ± SD (%)**
**Sample 1**	**Sample 2**	**Sample 3**			**Sample 1**	**Sample 2**	**Sample 3**	**Sample 1**	**Sample 2**	**Sample 3**	
–531	–531	–531	$V_2L_2^{2-}$	1–6	0.1	0.4	0.7	0.1	0.4	0.7	0.4 ± 0.3
–540	–539	–539	$V_3L_2^{3-}$ (or V_2L^{2-})	1–6	1.0	1.3	1.5	2.3	2.8	3.3	2.8 ± 0.5
–550	–549	–549	$V_3L_2^{3-}$ (or V_2L^{2-})	1–6	1.3	1.5	1.8				
–555	–555	–556	$[H_2V^VO_4]^-$	6,8	5.3	4.9	3.6	5.3	4.9	3.6	4.6 ± 0.9
–570	–570	–570	$[H_2V^V_2O_7]^{2-}$	6,8	5.1	5.2	4.5	5.1	5.2	4.5	4.9 ± 0.4
–574	–574	–574	$[V^V_4O_{12}]^{4-}$	6,8	70.3	70.7	70.0	70.3	70.7	70.0	70.3 ± 0.4
–582	–582	–582	$[V^V_5O_{15}]^{5-}$	6,8	16.9	16.0	17.9	16.9	16.0	17.9	16.9 ± 1.0
**5 mM $Cs_2[V^V_2O_4(lact)_2]\cdot 2H_2O$ + 13 mg/mL HEWL in 0.1 M HEPES, pH 7.5, 2.0 M sodium formate, 10% D_2O (after 7 d at RT)**											
**^{51}V chemical shift (ppm)**			**Species**	**References**	**Integration (%)**			**Sum**			**Average ± SD (%)**
**Sample 1**	**Sample 2**	**Sample 3**			**Sample 1**	**Sample 2**	**Sample 3**	**Sample 1**	**Sample 2**	**Sample 3**	

–531	–531	–531	$V_2L_2^{2-}$	1–6	0.5	0.6	0.4	0.5	0.6	0.4	0.5 ± 0.1
–539	–539	–539	$V_3L_2^{3-}$ (or V_2L^{2-})	1–6	1.3	1.9	1.4	3.2	4.5	3.1	3.6 ± 0.8
–549	–549	–549	$V_3L_2^{3-}$ (or V_2L^{2-})	1–6	1.9	2.6	1.7				
–556	–556	–556	$[H_2V^VO_4]^-$	6,8	5.3	6.3	4.7	5.3	6.3	4.7	5.4 ± 0.8
–570	–570	–570	$[H_2V^V{}_2O_7]^{2-}$	6,8	5.0	6.4	4.2	5.0	6.4	4.2	5.2 ± 1.1
–574	–574	–574	$[V^V{}_4O_{12}]^{4-}$	6,8	69.9	68.1	70.5	69.9	68.1	70.5	69.5 ± 1.2
–582	–582	–582	$[V^V{}_5O_{15}]^{5-}$	6,8	16.1	14.1	17.1	16.1	14.1	17.1	15.8 ± 1.5

[a] L indicates $lact^{2-}$ ligand, VL^- indicates $[V^VO_2(lact)(H_2O)]^-$, VL^{2-} $[V^VO_2(lact)(OH)]^{2-}$, $V_2L_2^{2-}$ $[V^V{}_2O_4(lact)_2]^{2-}$, V_2L^{2-} $[V^V{}_2O_5(lact)]^{2-}$ and $V_3L_2^{3-}$ $[V^V{}_3O_7(lact)_2]^{3-}$.

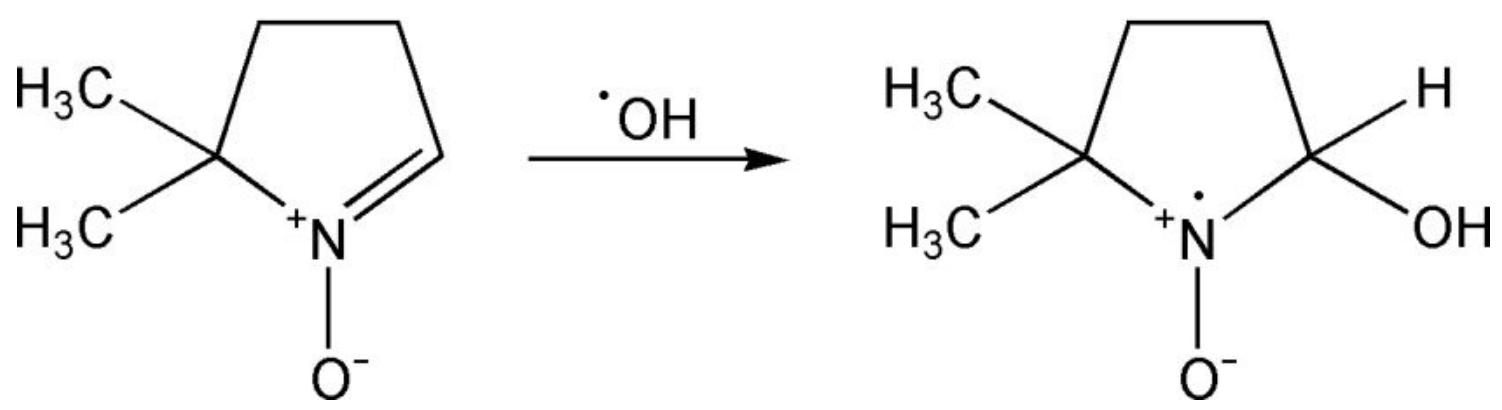

Figure S1. Adduct formation between the hydroxyl radical (•OH) and 5,5-dimethyl-1-pyrroline *N*-oxide (DMPO).

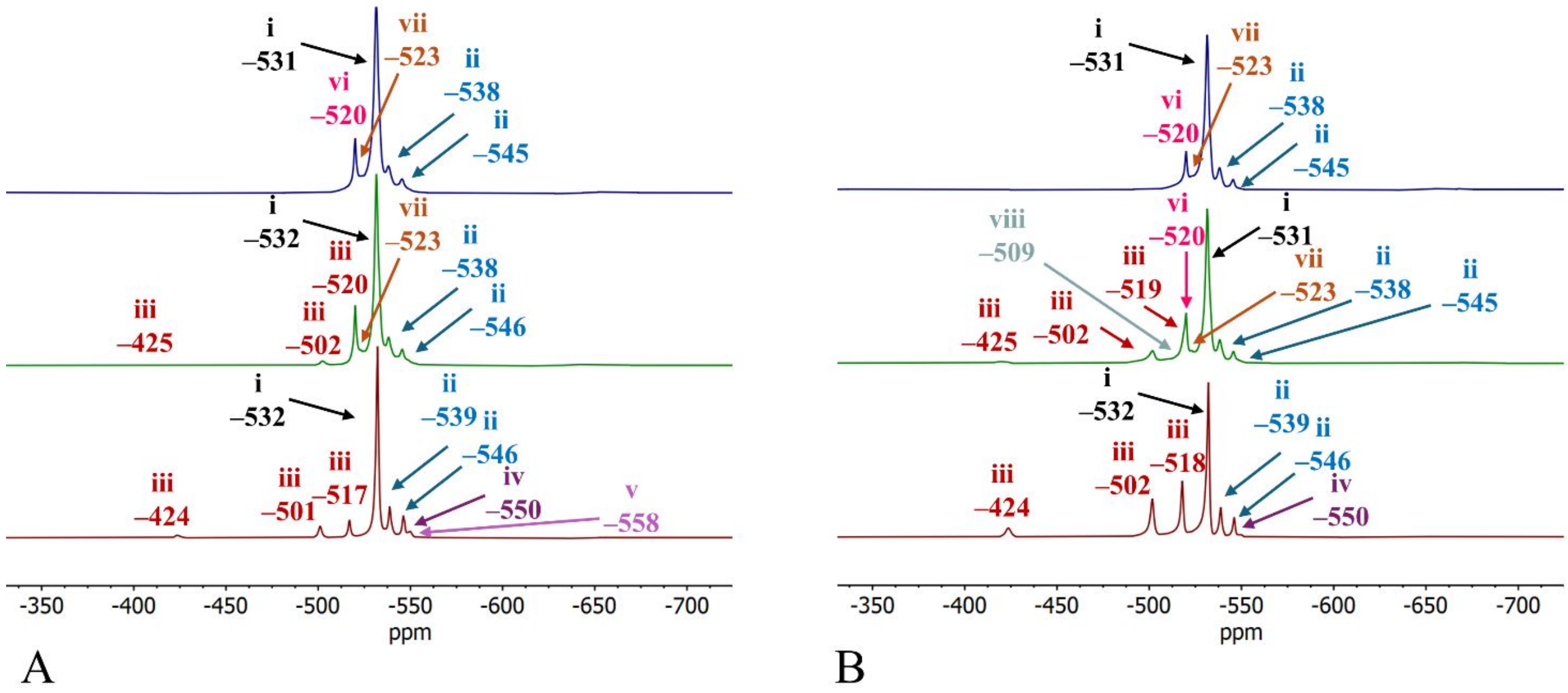

Figure S2. ^{51}V NMR spectra measured at RT after A) 1 h and B) 7 d of incubation of $Cs_2[V^V{}_2O_4(lact)_2]\cdot 2H_2O$ (5 mM) dissolved in D_2O (red lines); in 0.1 M sodium acetate, pH 4.0, 20% ethylene glycol, 0.6 M sodium nitrate, 10% D_2O (green lines); and in 0.1 M sodium acetate, pH 4.0, 20% ethylene glycol, 0.6 M sodium nitrate, 10% D_2O in presence of lysozyme (13 mg/mL) (blue lines). i indicates $[V^V{}_2O_4(lact)_2]^{2-}$, ii $[V^V{}_3O_7(lact)_2]^{3-}$, iii V_{10}, iv $[V^VO_2]^+$, v $[H_2V^VO_4]^-$, vi $[V^VO_2(lact)(OH)]^{2-}$, vii $[V^V{}_2O_4(EG)_2]^{2-}$ and viii $[V^VO_2(lact)(H_2O)]^-$.

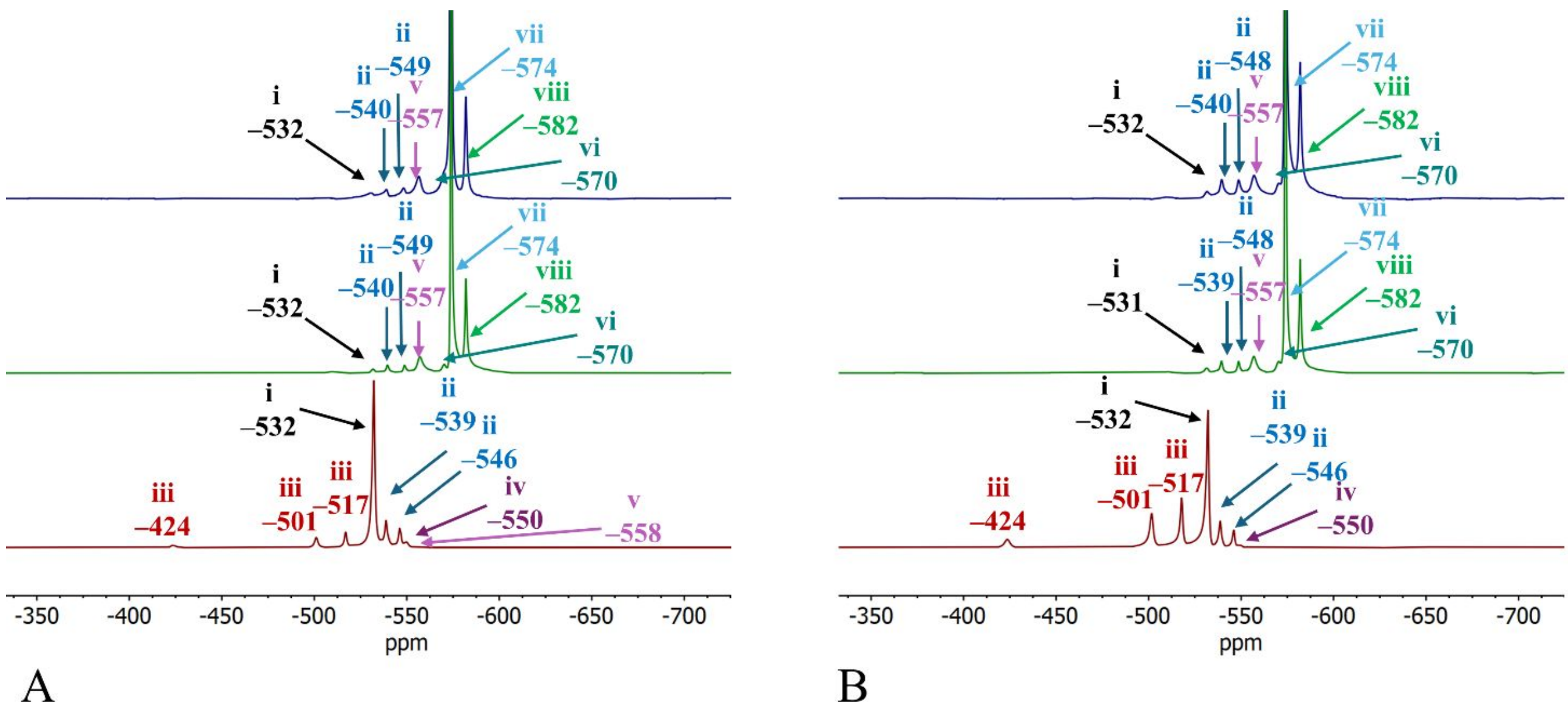

Figure S3. ^{51}V NMR spectra measured at RT after A) 1 h and B) 7 d of incubation of $Cs_2[V^V{}_2O_4(lact)_2]\cdot 2H_2O$ (5 mM) dissolved in D_2O (red lines); in 0.8 M succinic acid, pH 7.0, 10% D_2O (green lines); and 0.8 M succinic acid, pH 7.0, 10% D_2O in presence of lysozyme (13 mg/mL) (blue lines). i indicates $[V^V{}_2O_4(lact)_2]^{2-}$, ii $[V^V{}_3O_7(lact)_2]^{3-}$, iii V_{10}, iv $[V^VO_2]^+$, v $[H_2V^VO_4]^-$, vi $[H_2V^V{}_2O_7]^{2-}$, vii $[V^V{}_4O_{12}]^{4-}$ and viii $[V^V{}_5O_{15}]^{5-}$.

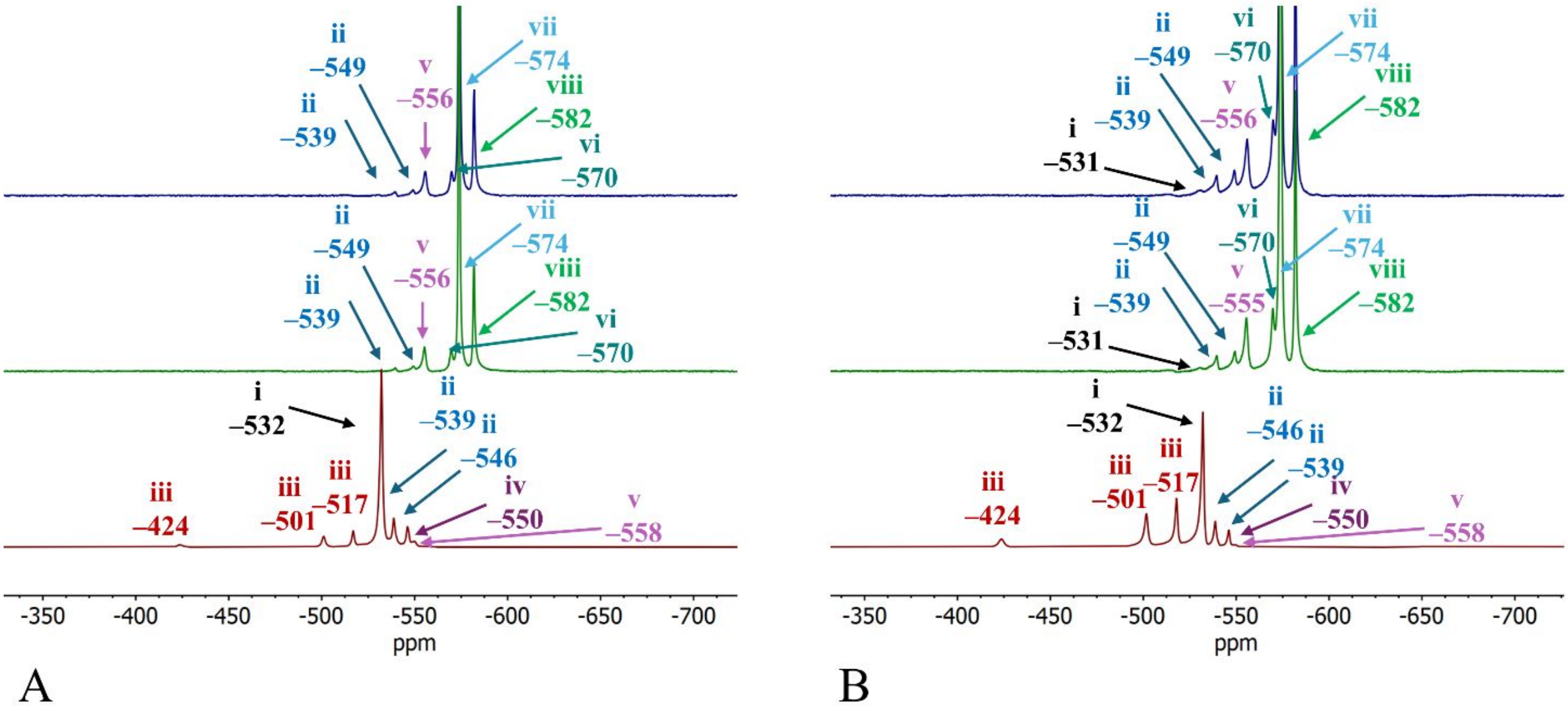

Figure S4. ^{51}V NMR spectra measured at RT after A) 1 h and B) 7 d of incubation of $Cs_2[V^V{}_2O_4(lact)_2]\cdot 2H_2O$ (5 mM) dissolved in D_2O (red lines); in 0.1 M HEPES, pH 7.5, 2.0 M sodium formate, 10% D_2O (green lines); and in 0.1 M HEPES, pH 7.5, 2.0 M sodium formate, 10% D_2O in presence of lysozyme (13 mg/mL) (blue lines). i indicates $[V^V{}_2O_4(lact)_2]^{2-}$, ii $[V^V{}_3O_7(lact)_2]^{3-}$, iii V_{10}, iv $[V^VO_2]^+$, v $[H_2V^VO_4]^-$, vi $[H_2V^V{}_2O_7]^{2-}$, vii $[V^V{}_4O_{12}]^{4-}$ and viii $[V^V{}_5O_{15}]^{5-}$.

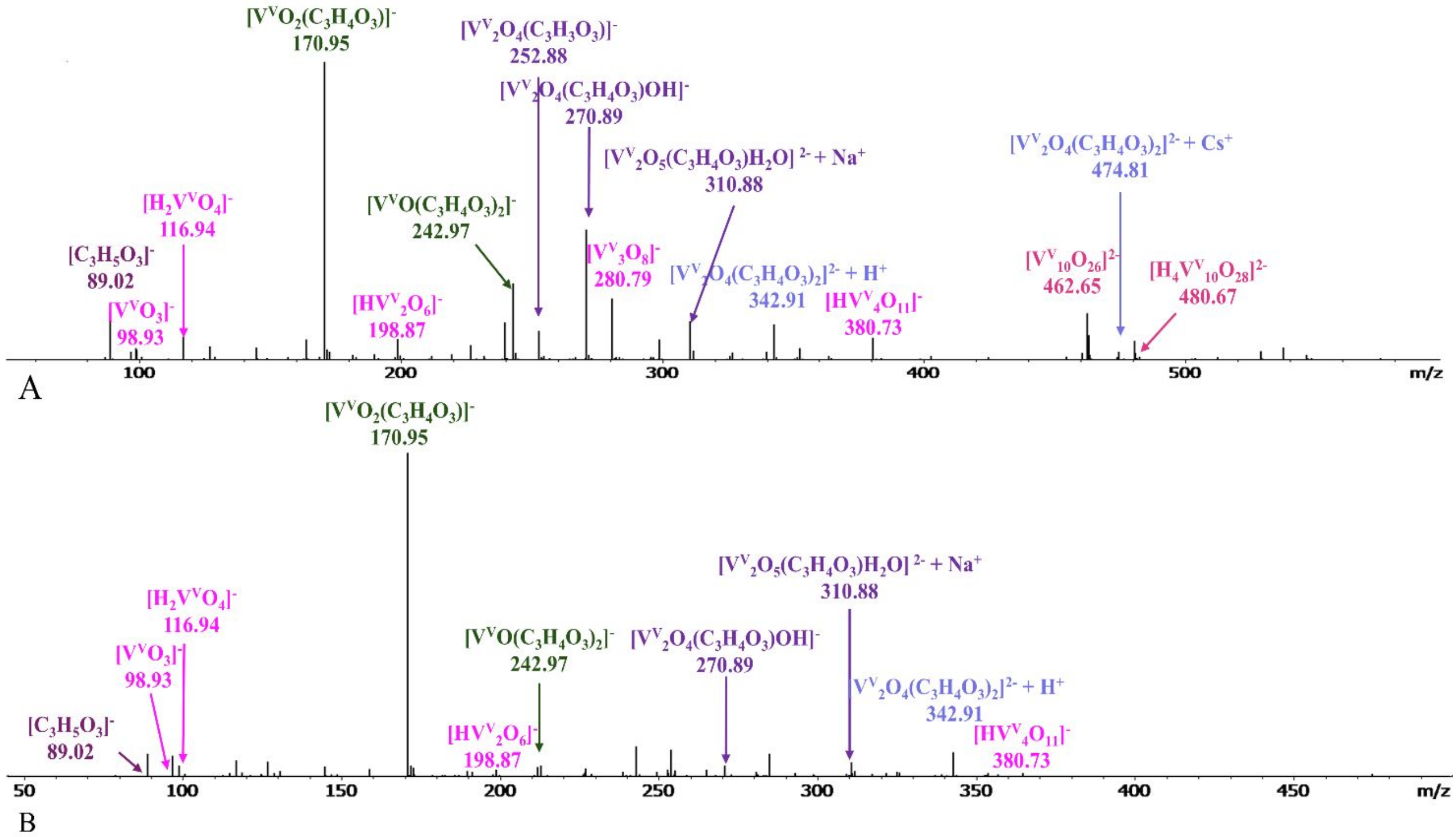

Figure S5. ESI mass spectrum of $Cs_2[V^V{}_2O_4(lact)_2]\cdot 2H_2O$ (~ 100 μM) in A) H_2O and in B) acetonitrile/methanol + 1% H_2O. The spectra were recorded in negative ion mode within the m/z range of 50 to 600, and the spectrometer was calibrated using the standard tunemix (Bruker Daltonik GmbH, timsTOF Pro User Manual, Bremen, Germany) to ensure an accuracy of approximately 5 ppm in the m/z region of 45-1800.

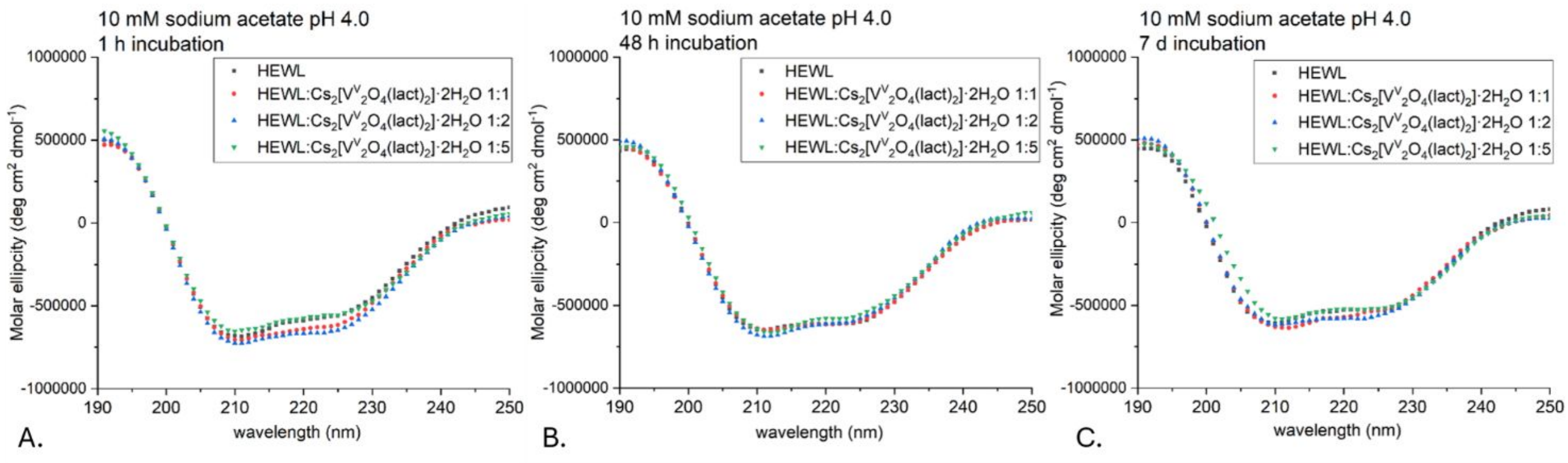

Figure S6. CD spectra of HEWL (7 μM in 0.01 M sodium acetate buffer, pH 4.0) in the absence (black squares) and in the presence of $Cs_2[V^V{}_2O_4(lact)_2]\cdot 2H_2O$ at 1:1 (red circles), 1:2 (blue triangles) and 1:5 (green triangles) protein to metal molar ratios upon (A) 1 h, (B) 48 h and (C) 7 days of incubation.

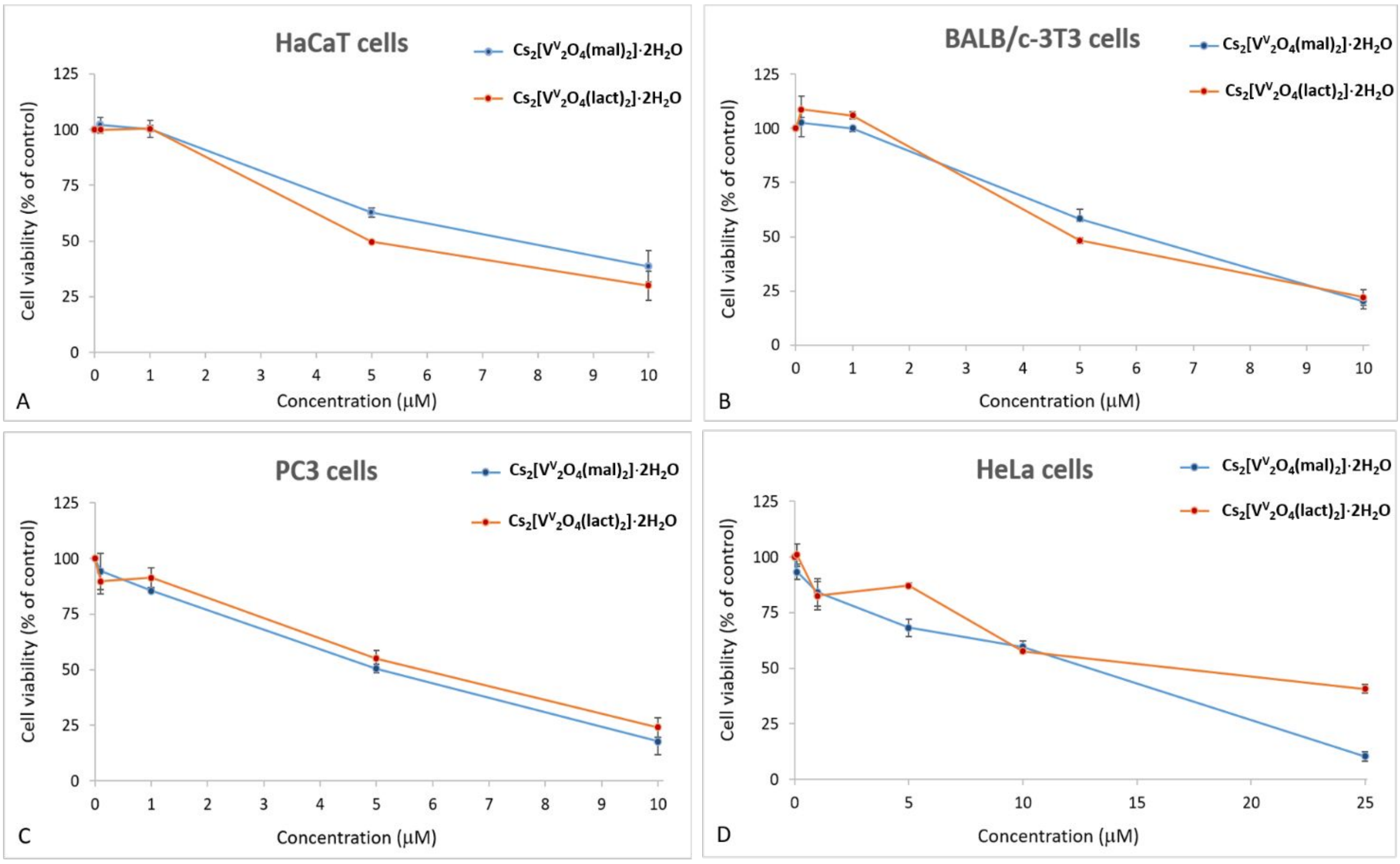

Figure S7. Effect of vanadium complexes on cell viability. Dose-response curves of HaCaT (A), BALB/c-3T3 (B), HeLa (B) and PC3 (D) incubated for 48 h with increasing concentrations of $Cs_2[V^V{}_2O_4(mal)_2]\cdot 2H_2O$ (blue curves) or $Cs_2[V^V{}_2O_4(lact)_2]\cdot 2H_2O$ (red curves). Cell viability was assessed by the MTT assay and cell survival expressed as percentage of viable cells in the presence of the complexes, with respect to control cells grown in the absence of the molecules.

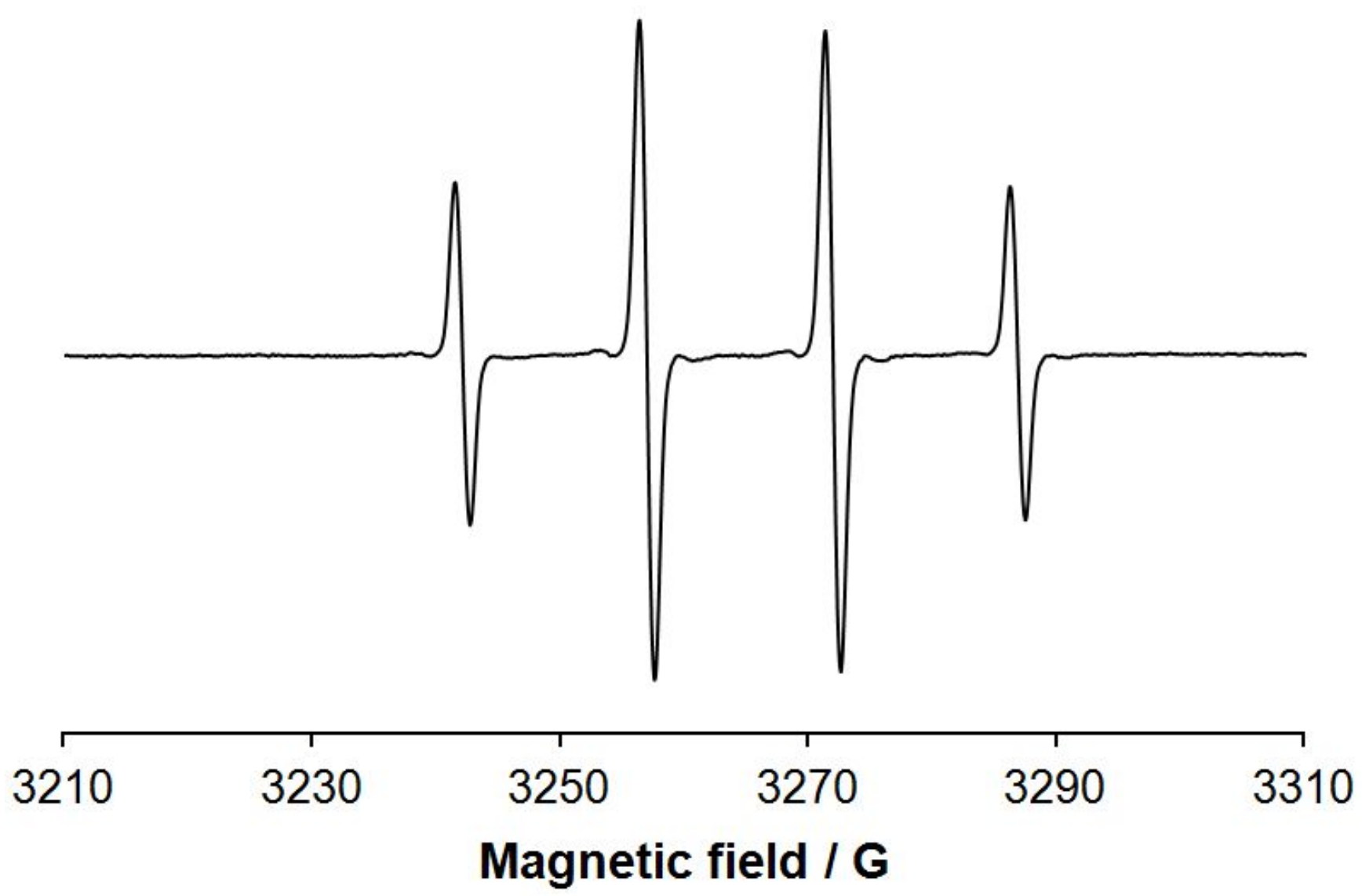

Figure S8. Experimental EPR spectrum of the adduct DMPO–OH. The ratio between the intensity of the four lines is 1:2:2:1.
